# Autoimmune Epilepsy - Novel Multidisciplinary Analysis, Discoveries and Insights

**DOI:** 10.3389/fimmu.2021.762743

**Published:** 2022-01-12

**Authors:** Mia Levite, Hadassa Goldberg

**Affiliations:** ^1^ Faculty of Medicine, The Hebrew University, Jerusalem, Israel; ^2^ Goldyne Savad Institute of Gene Therapy, Hadassah Hebrew University Hospital, Jerusalem, Israel; ^3^ Epilepsy Center, Schneider Children’s Medical Center of Israel, Petach Tikva, Israel; ^4^ Sackler Faculty of Medicine, Tel Aviv University, Tel Aviv, Israel

**Keywords:** epilepsy, autoimmune epilepsy, autoimmunity, glutamate receptor antibodies, GluR3B antibodies, HLA, T cells, neurological diseases

## Abstract

Epilepsy affects ~50 million people. In ~30% of patients the etiology is unknown, and ~30% are unresponsive to anti-epileptic drugs. Intractable epilepsy often leads to multiple seizures daily or weekly, lasting for years, and accompanied by cognitive, behavioral, and psychiatric problems. This multidisciplinary scientific (not clinical) ‘Perspective’ article discusses Autoimmune Epilepsy from immunological, neurological and basic-science angles. The article includes summaries and novel discoveries, ideas, insights and recommendations. We summarize the characteristic features of the respective antigens, and the pathological activity *in vitro* and in animal models of autoimmune antibodies to: Glutamate/AMPA-GluR3, Glutamate/NMDA-NR1, Glutamate/NMDA-NR2, GAD-65, GABA-R, GLY-R, VGKC, LGI1, CASPR2, and β2 GP1, found in subpopulations of epilepsy patients. Glutamate receptor antibodies: AMPA-GluR3 **B** peptide antibodies, seem so far as the most exclusive and pathogenic autoimmune antibodies in Autoimmune Epilepsy. They kill neural cells by three mechanisms: excitotoxicity, Reactive-Oxygen-Species, and complement-fixation, and induce and/or facilitate brain damage, seizures, and behavioral impairments. In this article we raise and discuss many more topics and new insights related to Autoimmune Epilepsy. 1. Few autoimmune antibodies tilt the balance between excitatory Glutamate and inhibitory GABA, thereby promoting neuropathology and epilepsy; 2. Many autoantigens are synaptic, and have extracellular domains. These features increase the likelihood of autoimmunity against them, and the ease with which autoimmune antibodies can reach and harm these self-proteins. 3. Several autoantigens have ‘frenetic character’- undergoing dynamic changes that can increase their antigenicity; 4. The mRNAs of the autoantigens are widely expressed in multiple organs outside the brain. If translated by default to proteins, broad spectrum detrimental autoimmunity is expected; 5. The autoimmunity can precede seizures, cause them, and be detrimental whether primary or epiphenomenon; 6. Some autoimmune antibodies induce, and associate with, cognitive, behavioral and psychiatric impairments; 7. There are evidences for epitope spreading in Autoimmune Epilepsy; 8. T cells have different ‘faces’ in the brain, and in Autoimmune Epilepsy: Normal T cells are needed for the healthy brain. Normal T cells are damaged by autoimmune antibodies to Glutamate/AMPA GluR3, which they express, and maybe by additional autoantibodies to: Dopamine-R, GABA-R, Ach-R, Serotonin-R, and Adrenergic-R, present in various neurological diseases (summarized herein), since T cells express all these Neurotransmitter receptors. However, autoimmune and/or cytotoxic T cells damage the brain; 9. The HLA molecules are important for normal brain function. The HLA haplotype can confer susceptibility or protection from Autoimmune Epilepsy; 10. There are several therapeutic strategies for Autoimmune Epilepsy.

## 1. Introduction

Epilepsy affects 1-2% of the world population. In about 30% of individuals with epilepsy, the etiology is unknown, after ruling out genetic mutations, severe injury and several other possible causes. In about 20-30% of epilepsy patients, anti-epileptic drugs (AED) fail to control the seizures. These patients often have multiple epileptic seizures daily or weekly, over the course of years.

Moreover, individuals with intractable epilepsy tend to present with severe additional neurological, cognitive, behavioral and psychiatric problems including: attention deficit hyperactive disorder, mood disorders, and abnormal learning and memory.

Autoimmunity Epilepsy was recognized, coined and discussed for the first time as an independent clinical entity, and as a possible direct cause of epilepsy of several types (not only as a secondary non-specific phenomenon accompanying seizures), in 2002, in Nature Immunology paper entitled “Autoimmune Epilepsy” written by ML (first author herein) (1). This paper was based on the pioneering published findings on Glutamate/AMPA GluR3 antibodies in Rasmussen’s Encephalitis (RE) – the first epilepsy type shown to be an Autoimmune Epilepsy, and later in patients with other types of severe and enigmatic intractable epilepsy (2–7).

During the last two decades, Autoimmune Epilepsy has become increasingly recognized as an independent clinical and scientific entity, and multiple original papers and reviews were published so far on this topic (many of which are cited in different chapters of this article).

Currently, an autoimmune cause of epilepsy is suspected mainly in the presence of frequent or medically intractable seizures and at least one neural autoimmune antibody, inflammatory changes indicated in serum or cerebrospinal fluid (CSF) or on MRI, or a personal or family history of autoimmunity (8, 11, 12).

The multiple scientific and clinical discoveries that support Autoimmune Epilepsy as a cause of epilepsy, derive from both *in vitro* and *in vivo* studies, on autoimmune antibodies of epilepsy patients, and on corresponding autoimmune antibodies produced in animal models [**Table 1** (1–16, 18, 21–26, 29–32, 35–37, 39–41, 45, 49)].

**Table 1:** The autoimmune antibodies, and the corresponding self-proteins/antigens in Autoimmune Epilepsy.

The autoimmune antibodies, and the self-proteins/antigens			Neurological diseases in which the autoimmune antibodies were found so far	Pathological activity of the autoimmune antibodies *in vitro*, and *in vivo* in animal models	
				*In vitro* evidence obtained in tissue culture	*In vivo* evidence obtained in animal models
The autoimmune antibodies and the self-protein/antigen they target	The subcellular location of the self-protein/antigen	The self-protein’s main function			
**Antibodies: GluR3B peptide antibodies**	Cell junction, Cell membrane, Postsynaptic cell membrane, Synapse	GluR3 is a key component - subunit of the AMPA type of ionotropic receptor for Glutamate - the major excitatory neurotransmitter in CNS.	Intractable chronic epilepsy, Autoimmune Epilepsy	GluR3 antibodies, and mainly GluR3 **B** peptide antibodies, induce multiple pathological effects *in vitro*. **1**. GluR3 antibodies, or GluR3 **B** peptide antibodies , whose origin in either epilepsy patients, mice, rats, or GluR3 **B** peptide monolclonal antibody (mAb), bind neural cells (2, 3, 5, 6, 10, 49, 82), **2**. GluR3, antibodies or GluR3 **B** peptide antibodies can activate AMPA receptors in neural cells (3, 6, 49, 82), and by doing so function as glutamate/AMPA agonists, without the requirement of neuronal, glial or blood ancillary molecules. **3.** GluR3 **B** peptide antibodies kill neural cells directly by excitotoxicity (6). **4**. GluR3 **B** peptide antibodies induce rapid production of Reactive Oxygen Species (ROS) in human neural cells, and then kill these cells (10). **5**. GluR3 antibodies destroy neural cells, first astrocytes and then neurons, in a complement-dependent manner, modulated by complement regulatory proteins (19). **6**. GluR3 **B** peptide antibodies bind, induce rapid ROS production) and then kill normal human T cells that express GluR3 (10).	IN RABBITS: Rabbits immunized with GluR3 developed GluR3 specific antibodies, and presented with anorexia and behavior characteristic of seizures, consisting of brief periods of immobilization, unresponsiveness, and repetitive clonic movements. The brain of the symptomatic GluR3-immunized rabbits disclosed inflammatory changes consisting of microglial nodules and perivascular lymphocytic infiltration (2, 3, 5). IN MICE: Model 1- Mice immunized with GluR3 **B** peptide developed both GluR3 **B** peptide specific antibodies, and GluR3 **B** peptide specific T cells, with significantly biased frequencies of particular TCR-Vβ families. The GluR3 **B**-immunized mice presented with multiple brain pathology, consisting of: **1.** Thickening of the cerebral meninges, **2.** Perivascular lympho-mononuclear cell Infiltration, **3.** Occasional (observed in few mice) pathologic gliosis in the cerebrum, **4.** Cerebellar cortical abiotrophy, with loss of neurons, from both the Purkinje and granule cell layers, **5.** Moderate to severe spongiform degeneration, mainly in the cortex, in the cerebellar white matter, and in few foci in the cerebrum and spinal cord (7).
**The self-protein/antigen: Glutamate receptor, AMPA type, subunit GluR3, peptide B, entitled GluR3B peptide**		GluR3 is expressed and functional also in non neural cells: T cells and various other cells.			
					Model 2- Epilepsy patient’s purified total IgG, rich in GluR3 **B** peptide antibodies, which was released continuously in normal mouse brain for 1 week, and followed continuously by video EEG for 2 weeks, induced all the following effects: **1.** Strong Seizures, **2.** Cerebellar Purkinje cell loss, **3.** Degeneration in the hippocampus and cerebral cortex, and **4.** Elevation of CD3^+^ T cells, and of activated Mac-2^+^ microglia and GFAP^+^ astrocytes in both the gray and white matter of the cerebral cortex, hippocampus, corpus calossum and cerebellum of mice (10).
					IN RATS: Rats immunized with GluR3 **B** peptide, developed GluR3 **B** peptide specific antibodies. Analysis of the rat's brains revealed several pathological features: **1.** Lower number of NeuN^+^ mature neurons in the superficial layer VI of the cortex, in both the motor cortex and the somatosensory cortex, which was accompanied by the appearance of more DCT^+^ positive newly born immature neurons in the subventricular zone, **2.** The GluR3 **B**-immunized rats had almost twice more GFAP^+^ astrocytes in both the motor cortex and the somatosensory cortex. The GFAP^+^ astrocytes had the characteristic reactive appearance, i.e. hypertrophy of their cellular processes, filled with increased amounts of GFAP, which is indicative of reactive gliosis (25).
**Antibodies:** **NR1 antibodies** **The self-protein/antigen: Glutamate receptor, NMDA type, subunit NR1, entitled NMDA-NR1**	Cell junction, Cell membrane, Postsynaptic cell membrane, Synapse	NR1 is is a key component - subunit of the NMDA type of ionotropic receptor for Glutamate – the major excitatory neurotransm itter in CNS. NR1 is expressed and functional also in non neural cells: T cells and many other cells.	NMDA Encephalitis, Intractable chronic epilepsy, Autoimmune Epilepsy	NMDA-NR1 antibodies decrease dramatically the level of synaptic NMDA receptors expressed on the cell surface, by crosslinking and internalization. They also decrease the levels of other synaptic proteins in neurons, along with prominent changes in NMDA receptor-mediated currents (147, 148). NMDA receptor antibodies also activate complement on NMDA receptor-expressing human embryonic kidney cells (63). The NR1 antibodies were functional in the NMDAR1 internalization assay based on human IPSC-derived cortical neurons (53). Despite this and surprisingly, NMDA-R1 antibodies were recently found to belong to the normal autoimmune repertoire of dogs, cats, rats, mice, baboons, and rhesus macaques (51). Since the age dependence of seroprevalence is lost in nonhuman primates in captivity and in human migrants, it is suggested that the NR1 antibodies may be related to chronic life stress (53).	**1.** Infusion of NMDR-NR1 antibodies into mouse brains could recapitulate encephalitis symptoms, while active immunization resulted also in strong T cell infiltration into the hippocampus [reviewed in (52, 53)]. **2.** In a murine model of in utero exposure to human recombinant NMDR-NR1 antibodies, pregnant mice were injected IP with human monoclonal antibodies. Offspring were investigated for acute and chronic effects on NMDAR function, brain development, and behavior. The transferred NR1 antibodies bound to synaptic structures in the fetal brain. The density of NMDAR was considerably reduced, and electrophysiological properties were altered, reflected by decreased amplitudes of spontaneous excitatory postsynaptic currents in the young neonates. The NMDA-NR1 antibody-treated animals displayed increased early postnatal mortality, impaired neurodevelopmental reflexes, altered blood pH, and reduced body weight. During adolescence and adulthood, animals showed hyperactivity, lower anxiety, and impaired sensorimotor gating. NMDA-NR1 antibodies caused long-lasting neuropathological effects also in aged mice, such as reduced volumes of cerebellum, midbrain, and brainstem (54). **3.** Upon injection of CSF of patients with Encephalitis, that contained anti-NMDA-NR1/NR2 heterodimer antibodies, or of IgG purified from the patient’s sera, into rat’s brain, there was an increase in the afferent facilitation of corticomotor responses (149). **4.** NMDA-NR1 antibodies of the IgG class shape behavioral phenotypes upon access to the brain, but do not cause brain inflammation on their own (51).
**Antibodies: NR2 antibodies** **The self protein/antigen: Glutamate receptor: NMDA type, subunit NR2, entitled NMDA-NR2**	Cell junction, Cell membrane, Membrane, Postsynaptic cell membrane, Synapse	NR2 is a key component - subunit of the NMDA type of ionotropic receptor for Glutamate – the major excitatory neurotransmitter in CNS.	Neuropsychiatric SLE, Paraneoplastic Encephalitis, Mania, Schizophrenia, Slowly progressive cognitive impairment & other?	The NMDA-NR2A/NR2B antibodies are pathogenic and neurotoxic in vitro and in vivo, as proven by multiple studies, among them (89–92, 150–153, 157, 158, 162, 163). In lupus-prone mice, when NMDA-NR2A/NR2B antibodies gain access to the brain, they induce neurotoxic effects: they cause neuronal death in vivo, with ensuing cognitive dysfunction and emotional disturbance. The NMDA-NR2 antibodies induce in vivo neuropsychopathology: either impaired memory and hippocampal atrophy, or emotional disturbances and atrophy of the amygdala, that followed the neuronal death [for reviews see (90, 92)]. See also above the cited papers with regards to the NMDA-NR1 antibodies, some of which are also relevant also to NMDA-NR2 antibodies.	
**Antibodies:** **GAD65 antibodies** **The self-protein/antigen: Glutamic acid decarboxylase** **(GAD) 65**	Cell membrane, Cell projection, Synapse, Cell junction, Cytoplasmic vesicles, Golgi apparatus	GAD65 is an intracellular enzyme that catalyze the production of GABA: Catalyzes the decarboxy lation of Glutamate to GABA and CO2	Limbic encephalitis, Intractable chronic epilepsy, Autoimmune Epilepsy, Stiff person syndrome	CSF immunoglobulins prepared from a patient with cerebellar ataxia associated with GAD65 antibodies suppressed GABA-mediated transmission on cerebellar Purkinje cells. The IgG acted on the presynaptic terminals of GABAergic interneurons and decreased GABA release onto Purkinje cells. These inhibitory effects were most likely elicited by the GAD65 antibodies (44) (15).	1. Intracerebellar administration of IgG from patients with GAD antibodies and neurological involvement (IgG-GAD) into rats, blocked the potentiation of the corticomotor response normally associated with trains of repetitive peripheral nerve stimulation. When injected in the lumbar paraspinal region, the IgG-GAD induced continuous motor activity with repetitive discharges, abnormal exteroceptive reflexes, and increased excitability of anterior horn neurons. Furthermore, IgG-GAD significantly reduced the NMDA-mediated production of nitric oxide in cerebellar nuclei, and impaired the synaptic regulation of glutamate after NMDA administration. These effects were not observed after administration of IgG from the following 2 groups: 1. patients with GAD antibodies and diabetes mellitus, but without neurological complications; and 2. control patients (141). 2. Human Stiff person syndrome (SPS) IgG-containing high titer GAD65 antibodies induce motor dysfunction in rats (66). Specifically, IgG of an SPS patient with severe motor impairment but without anxious comorbidity, containing high titers of GAD65 antibodies was injected into the lateral ventricle or intrathecally, at the spinal level of experimental rats. The injected rats showed stiffness-like behavior with impaired walking ability, and reduced grip strength of the upper limbs, as well as postural and sensorimotor dysfunction (66).
**Antibodies:** **GABA-R antibodies** **The self-protein/antigen: GABA-B receptor**, **B1 subunit**	Cell junction, Cell membrane, Synapse	GABA-R is a ligand-gated chloride channel receptor for GABA - the major inhibitory neurotransmitter in the brain. GABA-R is expressed and functional also in T cells, and various other cells.	SLE, Encephalitis, Intractable chronic epilepsy, Autoimmune Epilepsy	Immunoprecipitation and mass spectrometry showed that patients with encephalitis suspected to be paraneoplastic or immune mediated, have antibodies that recognize the B1 subunit of the GABA(B) receptor. Confocal microscopy showed co- localisation of the antibody with GABA(B) receptors (60). Acute application of patient’s IgG containing GABA-B receptor antibodies to primary hippocampal neuronal cultures decreased both the duration of network UP states, and the spike rate of pyramidal cells in the entorhinal cortex. GABA-B receptor antibodies do not appear to affect GABA-B receptors by internalization, but rather reduce excitability on the medial temporal lobe networks (67).	Purified IgG that contain GABA(B) receptor antibodies, of a patient with recurrent acute episodes of respiratory crises, autonomic symptoms and total insomnia (agrypnia), and GABA(B)R1 antibodies, were injected intrathecally into cisterna magna of normal mice pre-implanted with EEG electrodes. Following this injection, severe ataxia, followed by breathing depression and total suppression of slow wave sleep, as evidenced by EEG recording, were observed (170). Immunohistochemistry on brain sections of the mice injected with the patient’s IgG showed the simultaneous presence of bound human IgG and C5b-9 deposits on Purkinje cells and cerebellar granular layer. After incubation with the GABA(B)R antibodies, a marked reduction of receptor immunostaining was found with relative sparing of neuronal architecture (170).
**Antibodies:** **GLYR antibodies** **The self-protein/antigen:** **Glycine receptor**	Cell junction, Cell membrane, Cell projection, Membrane, Postsynaptic cell membrane, Synapse, Perikaryon, Dendrite	Glycine receptor (GlyR) is a an ionotropic receptor of Glycine - an inhibitory neurotransmitter in the central nervous system, especially in the spinal cord, brainstem, and retina. GlyR down regulate neuronal excitability, and neuro- transmission in the spinal cord. GlyR play a crucial role in nociceptive signaling and in multiple motor and sensory functions	Brainstem disorders. mainly in patients with progressive encephalomyelitis with rigidity and myoclonus. Stiff person syndrome plus, or progressive encephalomyelitis with rigidity and myoclonus (PERM).	GlyR antibodies activated complement on the cell surface of live GlyR-1 expressing HEK cells, at room temperature, and caused internalization and lysosomal degradation of the glycine receptors at 37°C (69).	GlyR IgG injected mice showed impaired ability on the rotarod from days 5 to 10, but this was normalized by day 12. No other behavioural differences were documented, but the GlyR IgG-injected mice had IgG deposits on neurons that express GlyRs in the brainstem and spinal cord. The IgG was not only on the surface, but also inside these large GlyR-expressing neurons, which continued to express surface GlyR (74).
**Antibodies:** **VGKC antibodies** **The self-protein/antigen: Voltage gated potassium** **channels, mainly Kv** **1.1, 1.2 or 1.6**	Cell membrane, Cell projection, Membrane	Voltage-gated potassium channels, expressed in many cells, mediate transmembrane potassium transport, and contribute to the regulation of the membrane potential.	It is claimed that VGKC antibodies do not indicate a specific clinical syndrome, and that they are nonspecific biomarkers of inflammatory neurologic diseases, particularly of encephalopathy (34). Present in some patients with encepsalitis, intractable and chronic epilepsy, and Autoimmune Epilepsy. Common also in healthy controls	? Is there direct compelling evidence for the pathogenicity of VGKC antibodies in vitro? See discussion and conclusions in (34). Some VGKC antibodies may bind to intracellular epitopes on the VGKC subunits, or to the intracellular interacting proteins, but for many the targets remain undefined (34).	? Is there direct and compelling evidence for the pathogenicity of VGKC antibodies *in vivo*, in animal models? See discussion and conclusions in (34). Interestingly, evidence have been published showing that Kv1.1 antibodies may have protective anti-convulsive effects in a mouse model of lithium-pilocarpine temporal lobe epilepsy. Treatment of epileptic mice with VGKC antibodies for 30 days decreased neuronal loss in structures classically associated with attentional performance in hippocampus. The mice treated with VGKC antibodies had also inhibited motor seizures and hippocampal damage, compared with control mice. It is suggested that VGKC may even be a potential target for the treatment of epilepsy (171).
**Antibodies:** **LGI1 antibodies** **The self-protein/antigen:** **Glioma-inactivated 1** **(LGI1) protein**	Cell junction, Secreted, Synapse	LGI1 is a secreted neuronal protein that regulates VGKCs. It forms a trans-synaptic complex that includes the presynaptic disintegrin ADAM23, which interacts with Kv1.1 VGKCs, and postsynaptic ADAM22, which interacts with Glutamate/AMPA receptors.	Limbic encephalitis, Morvan’s syndrome, Peripheral nerve hyperexcitability,	1. IgG of patients with Limbic encephalitis, but not from healthy participants, prevent the binding of LGI1 to ADAM23 and ADAM22 (70). LGI1- and CASPR2 antibodies containing CSFs, of patients with autoantibody-mediated forms of encephalitis, increased the probability of glutamate release from CA3 neurons. In addition, these CSFs induced epileptiform activity at a population level following Schaffer collateral stimulation (169).	Cerebroventricular transfer of IgG of patients with anti-LGI1 associated limbic encephalitis into mice, induced all the following effects and others: A. Decreased the total and synaptic levels of Kv1.1 and AMPA receptors, B. Induced neuronal hyperexcitability with increased glutamatergic transmission, and higher presynaptic release probability, C. Impaired synaptic plasticity, D. Induced severe memory deficits (70).
**Antibodies:** **CASPR2 antibodies** **The self-protein/antigen:** **Contactin-associated** **protein 2 (CASPR2)**		CASPR2 is required, with CNTNAP1, for the formation of functional distinct domains critical for saltatory conduction of nerve impulses in myelinated nerve fibers. It demarcates the juxtap-aranodal region of the axo-glial junction. CASPR2 is a part of the VGKC complex.	Limbic encephalitis, Morvan’s syndrome, Peripheral nerve hyperexcitabili ty	1. CASPR2 antibodies bind to hippocampal neurons and to CASPR2-transfected HEK cells, and lead to some internalization of the IgG (72). CASPR2 and LGI1 antibodies containing CSFs, of patients with autoantibody-mediated forms of encephalitis, increased the probability of glutamate release from CA3 neurons. In addition, these CSFs induced epileptiform activity at a population level following Schaffer collateral stimulation (169). The CASPR2 antibodies containing CSF was also associated with higher spontaneous firing of CA1 pyramidal neurons (169).	**1.** Mice exposed IP to patient-derived CASPR2 IgG, displayed reduced working memory during the continuous spontaneous alternation test, with trends towards reduced short-term and long-term memories (72). CASPR2-IgG injected mice showed longer latency to start interacting, associated with more freezing behavior, and reduced non-social activities of rearing and grooming. Moreover, in their brains there was increased c-fos expression in the piriform-entorhinal cortex and hypothalamus, and a modest loss of Purkinje cells (141). **2.** Mice offsprings exposed to patient’s-derived CASPR2 antibodies (IgG) in utero, expressed persistent microglial activation and synaptic loss with behavioral abnormalities. The CASPR2-IgG exposed progeny showed marked social interaction deficits, abnormally located glutamatergic neurons in layers V-VI of the somatosensory cortex, a 16% increase in activated microglia, and a 15-52% decrease in glutamatergic synapses in layers of the prefrontal and somatosensory cortices (73).

The Table shows the main types of autoimmune antibodies found in subpopulations of epilepsy patients, the other neurological diseases in which they are found in addition to epilepsy, the self-proteins/antigens targeted by the autoimmune antibodies, and the pathological activity in vitro, and in vivo in animal models, of these autoimmune antibodies, discovered so far.

Immunotherapy for Autoimmune Epilepsy, which was first proposed in (17), and has been discussed in many publications since [for example (8, 12, 18)], has demonstrated effectiveness in some patients. See Part 14 and the papers cited therein, dealing with the current therapeutic strategies for Autoimmune Epilepsy.

While Autoimmune Epilepsy is increasingly accepted and diagnosed, many issues are still mysterious, many questions are still open, and several novel out of the box scientific discoveries have been published in the last few years. All of these require fresh analysis, review and discussion.

In addition, several novel interdisciplinary hypotheses raised in this paper deserve to be taken into account and researched in depth.

## 2. Twelve Types of Autoimmune Antibodies Have Been Detected So Far in the Serum and/or CSF of Subpopulations of Epilepsy Patients, and of Other Patients With Seizures and Encepahlitis. More May Be Discovered in the Future

At least 12 autoimmune antibodies have been found in various subpopulations of individuals with intractable epilepsy, or with encephalitis and seizures (2–24, 26–31, 35–37, 39–42, 44–47, 55).

**Figure 1:**
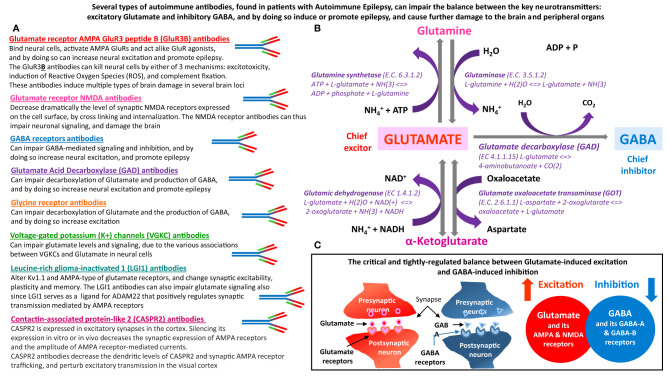
The main types of autoimmune antibodies present in subpopulations patients with Autoimmune Epilepsy can impair the balance between excitatory Glutamate and inhibitory GABA, and by doing so induce or promote epilepsy. These antibodies can also induce many other pathological effects in both the brain and peripheral organs, that express Glutamate and GABA receptors, and that depend on normal carefully-regulated levels and effects of Glutamate and GABA. **(A)** Several types of autoimmune antibodies present in some epilepsy patients, can directly or indirectly affect the very delicate and tightly-regulated balance between Glutamate – the chief excitatory neurotransmitter in the nervous system, and inhibitory GABA – the chief inhibitory neurotransmitter in the nervous system. By doing so, these autoimmune antibodies can trigger and/or promote epilepsy and many other pathological effects in the brain and in peripheral organs. The autoimmune antibodies, that can impair the balance between Glutamate and GABA are: Glutamate receptor, AMPA type, subunit GluR3, peptide B (GluR3 **B** ) antibodies, Glutamate receptor, NMDA type, subunit NR1 and/or NR2 antibodies, GABA receptors antibodies, Glutamate Acid Decarboxylase (GAD) antibodies, Glycine receptor antibodies, Voltage-gated potassium (K^+^) channels (VGKC) antibodies, Leucine-rich glioma-inactivated 1 (LGI1) antibodies, and Contactin-associated protein-like 2 (CASPR2) antibodies. The text below the name of these autoimmune antibodies, summarizes the main ways by which they can impair the balance between the levels, signaling and activity of either Glutamate and Glutamate receptors, or GABA and GABA receptors. **(B)** Glutamate and GABA biosynthesis pathways, and all the involved enzymes. Glutamate is the metabolic precursor of GABA, which can be recycled through the tricarboxylic acid cycle to synthesize Glutamate. GABA is formed from Glutamate by the action of Glutamate decarboxylase. **(C)** Schematic representation of the balance between Glutamate-induced neural excitation and GABA-induced neural inhibition.

These autoimmune antibodies are specified in the text below. In addition, the respective antigens, characteristic features and pathogenic activity in vitro and in vivo of most of these autoimmune antibodies are summarized in **Table 1**. Additional information about them is shown in **Tables 2** and **3**, and in **Figure 1**.

**Table 2:** The genes, main protein function, and mRNA and protein expression of the key self-proteins/antigens, which are targeted by different autoimmune antibodies in Autoimmune Epilepsy.

GLUTAMATE RECEPTOR - AMPA TYPE SUBUNIT 3 - GluR3
The gene: GRIA3. Main function of protein: Key subunit of AMPA ionotropic receptor for Glutamate -the major excitatory neurotransmitter in the nervous system. Main protein expression known so far: Brain, Endocrine tissues, Male tissues, Female tissues, Adipose & soft tissue, T cells {for GluR3 expression in human T cells see [(78, 79, 80, 10, 102, 103, 116), and for reviews on GluR3 expression and glutamate-induced effects on T cells see (100, 103)]}.. RNA expression in organs and tissues: Brain: Cerebral cortex, Olfactory region, Hippocampal formation, Amygdala, Basal ganglia, Thalamus, Hypothalamus, Midbrain, Pons and medulla, Cerebellum, Corpus callosum, Spinal cord; Eye: Retina; Endocrine tissues: Thyroid gland, Parathyroid gland, Adrenal gland, Pituitary gland; Muscle: heart muscle, smooth muscle, skeletal muscle; Lymphoid tissues and organs: Thymus, Appendix, Spleen, Lymph node, Tonsil, Bone marrow; Heart; Gastrointestinal tract: Stomach, Duodenum, Small intestine, Colon, Rectum; Proximal digestive tract: Salivary gland and Esophagus; Lung; Liver and Gallbladder; Pancreas; Kidney and Urinary bladder; Skin; Blood (mainly in T cells); Adipose and fat tissues; Female tissues: Ovary, Vagina, Fallopian tube, Endometrium, Cervix, Uterine, Placenta, and Breast; Male tissues: Testis, Epidydimis, Seminal vesicle and Prostate. Main data sources: https://www.proteinatlas.org/ENSG00000125675-GRIA3/tissue; https://www.uniprot.org/uniprot/P42263#expression
GLUTAMATE RECEPTOR – NMDA TYPE SUBUNITS NR1 AND NR2
The genes: GRIN1, GRIN2A, GRIN2B, GRIN2D; Main function of protein: Key subunit of NMDA ionotropic receptor for Glutamate -the major excitatory neurotransmitter in the nervous system. Main protein expression known and studied so far: Brain, Endocrine tissues, Lung, Gastrointestinal tract, Liver, Kidney & Urinary bladder, Male tissues, Female tissues, Appendix, T cells [for expression of NMDA receptors in human T cells T cells see for example (117, 173–176, 10)]. RNA expression in organs and tissues: Brain: Cerebral cortex, Olfactory region, Hippocampal formation, Amygdala, Basal ganglia, Thalamus, Hypothalamus, Midbrain, Pons and medulla, Cerebellum, Corpus callosum, Spinal cord; Eye: Retina; Endocrine tissues: Thyroid gland, Parathyroid gland, Adrenal gland, Pituitary gland; Muscle tissues: Heart muscle, Smooth muscle, Skeletal muscle; Lymphoid tissues and organs: Thymus, Appendix, Spleen, Lymph node, Tonsil, Bone marrow; Lung: Nasopharynx, Bronchus; Proximal digestive tract: Tongue, Oral mucosa, Salivary gland, Esophagus; Gastrointestinal tract: Stomach, Duodenum, Small intestine, Colon, Rectum; Liver and Gallbladder; Pancreas; Kidney & Urinary bladder; Male tissues: Ductus deferens, Testis, Epididymis, Seminal vesicle, Prostate; Female tissues: Vagina, Ovary, Fallopian tube, Endometrium, Cervix, Uterine, Placenta, Breast; Adipose & soft tissue; Skin; Blood: Mainly in T cells and Dendritic cells.
MAIN DATA SOURCES: https://www.uniprot.org/uniprot/Q05586#expression; https://www.proteinatlas.org/ENSG00000176884-GRIN1/tissue; https://www.proteinatlas.org/ENSG00000105464-GRIN2D/tissue
GABA RECEPTORS
The gene: GABRA1. Main function of protein: Ligand-gated chloride channel receptor for GABA, the major inhibitory neurotransmitter in the brain. Main protein expression known so far: Brain - Cerebral cortex, Cerebellum, Hippocampus, Caudate, and T cells [For T cells see for example (120, 121)].
RNA expression in organs and tissues: Brain: Cerebral cortex, Olfactory region, Hippocampal formation, Amygdala, Basal ganglia, Thalamus, Hypothalamus, Midbrain, Pons and medulla, Cerebellum, Corpus callosum, Spinal cord; Eye: Retina; Endocrine tissues: Thyroid gland, Parathyroid gland, Adrenal gland; Muscle tissues: Heart muscle, Smooth muscle, Skeletal muscle; Bone marrow & lymphoid tissues: Appendix, Spleen, Lymph node, Tonsil, Bone marrow; Lung; Proximal digestive tract: Salivary gland, Esophagus; Gastrointestinal tract: Stomach, Duodenum, Small intestine, Colon, Rectum; Liver & Gallbladder; Pancreas; Kidney & Urinary bladder; Male tissues: Testis, Epididymis, Seminal vesicle, Prostate; Female tissues: Vagina, Ovary, Fallopian tube, Endometrium, Cervix, Uterine, Placenta, Breast; Adipose & soft tissue; Skin; Blood – none of the cells tested. Main data source: https://www.proteinatlas.org/ENSG00000022355-GABRA1/tissue, and few related research papers.
GLUTAMIC ACID DECARBOXYLASE (GAD)
The gene: GAD2 (GAD65). Main function of protein: The enzyme that catalyzes the production of GABA- the primary inhibitory neurotransmitter in the brain, and a major inhibitory neurotransmitter in the spinal cord. GAD catalyzes the decarboxylation of glutamate to GABA and CO_2_. Main protein expression known so far: Brain - Cerebral cortex, Cerebellum, Hippocampus, Caudate, and Pancreas. RNA expression in organs and tissues: Brain: Cerebral cortex, Olfactory region, Hippocampal formation, Amygdala, Basal ganglia, Thalamus, Hypothalamus, Midbrain, Pons and medulla, Cerebellum, Corpus callosum, Spinal cord; Eye: Retina; Endocrine tissues: Thyroid gland, Parathyroid gland; Muscle tissues: Heart muscle, Smooth muscle, Skeletal muscle; Bone marrow & lymphoid tissues: Appendix, Spleen, Lymph node, Tonsil, Bone marrow; Lung; Proximal digestive tract; Gastrointestinal tract: Stomach, Duodenum, Small intestine, Colon, Rectum; Liver & Gallbladder; Pancreas; Kidney & Urinary bladder; Male tissues: Testis, Epididymis, Seminal vesicle, Prostate; Female tissues: Ovary, Fallopian tube, Endometrium, Cervix, Uterine, Placenta, Breast; Adipose & soft tissue: Skin.
MAIN DATA SOURCES: https://www.uniprot.org/uniprot/Q05329; https://www.proteinatlas.org/ENSG00000136750-GAD2/tissue
VOLTAGE-GATED POTASSIUM CHANNELS
The gene: KCNA1, KCNA2, KCNA4, KCNA5, KCNA6, KCNA7. Main function of protein: Mediates transmembrane potassium transport across membranes in excitable cells, and thereby contributes to the regulation of the membrane potential and nerve signaling. Expressed in brain. Highly expressed also in T cells, and at lower levels in the testis, lung, kidney, colon and heart. Main protein expression known so far: Display broad distributions in the nervous system and several other organs and also in T cells [see (172)]. RNA expression in organs and tissues: Brain: Cerebral cortex, Olfactory region, Hippocampal formation, Amygdala, Basal ganglia, Thalamus, Hypothalamus, Midbrain, Pons and medulla, Cerebellum, Corpus callosum, Spinal cord; Eye: Retina; Endocrine tissues: Thyroid gland, Parathyroid gland, Adrenal gland, Pituitary gland; Muscle tissues: Heart muscle, Smooth muscle, Skeletal muscle; Bone marrow & lymphoid tissues: Appendix, Spleen, Lymph node, Tonsil, Bone marrow; Lung: Lung, Bronchus; Proximal digestive tract: Tongue, Oral mucosa, Salivary gland, Esophagus ; Gastrointestinal tract: Stomach, Duodenum, Small intestine, Colon, Rectum; Liver & gallbladder; Pancreas; Kidney & urinary bladder; Male tissues: Testis, Epididymis, Seminal vesicle, Prostate; Adipose & soft tissue; Skin; Blood: B cells, T cells.
Main data source: https://www.proteinatlas.org/ENSG00000111262-KCNA1/tissue.
LEUCINE-RICH GLIOMA-INACTIVATED 1 (LGI1)
The gene: LGI1. Main function of protein: Regulates voltage-gated potassium channels assembled from KCNA1, KCNA4 and KCNAB1. It slows down channel inactivation by precluding channel closure mediated by the KCNAB1 subunit. Ligand for ADAM22 that positively regulates synaptic transmission mediated by Glutamate receptors of the AMPA type.
Main protein expression known so far: Neural tissues, especially in brain. RNA expression in organs and tissues: Brain: Cerebral cortex, Olfactory region, Hippocampal formation, Amygdala, Basal ganglia, Thalamus, Hypothalamus, Midbrain, Pons and medulla, Cerebellum, Corpus callosum, Spinal cord; Eye: Retina; Endocrine tissues: Thyroid gland, Parathyroid gland, Adrenal gland, Pituitary gland; Muscle tissues: Heart muscle, Smooth muscle, Skeletal muscle; Bone marrow & lymphoid tissues: Appendix, Spleen, Lymph node, Tonsil, Bone marrow; Lung; Proximal digestive tract: Salivary gland, Esophagus; Gastrointestinal tract: Stomach, Duodenum, Small intestine, Colon, Rectum; Liver & gallbladder; Pancreas; Kidney & urinary bladder; Male tissues: Testis, Epididymis, Seminal vesicle, Prostate; Female tissues: Ovary, Fallopian tube, Endometrium, Cervix, Uterine, Placenta, Breast; Adipose & soft tissue; Skin.
Main data sources: https://www.proteinatlas.org/ENSG00000108231-LGI1/tissue; https://www.uniprot.org/uniprot/O95970
CONTACTIN-ASSOCIATED PROTEIN-LIKE 2 (CASPR2)
The gene: CNTNAP2. Main function of protein: CASPR2 is required, with CNTNAP1, for the formation of functional distinct domains critical for saltatory conduction of nerve impulses in myelinated nerve fibers. It demarcates the juxtaparanodal region of the axo-glial junction. Main protein expression known so far: Distinct expression in the CNS, in multiple brain regions: RNA expression in organs and tissues: Brain: Cerebral cortex, Olfactory region, Hippocampal formation, Amygdala, Basal ganglia, Thalamus, Hypothalamus, Midbrain, Pons and medulla, Cerebellum, Corpus callosum, Spinal cord; Eye: Retina; Endocrine tissues: Thyroid gland, Parathyroid gland, Adrenal gland, Pituitary gland; Muscle tissues: Heart muscle, Smooth muscle, Skeletal muscle; Bone marrow & lymphoid tissues: Thymus, Appendix, Spleen, Lymph node, Tonsil, Bone marrow; Lung; Proximal digestive tract; Gastrointestinal tract: Stomach, Duodenum, Small intestine, Colon, Rectum; Liver & gallbladder; Pancreas; Kidney & urinary bladder; Male tissues: Testis, Epididymis, Seminal vesicle, Prostate; Female tissues: Vagina, Ovary, Fallopian tube, Endometrium, Cervix, Uterine, Placenta, Breast; Adipose & soft tissue; Skin; Blood: B cells.
Main data source: https://www.proteinatlas.org/ENSG00000174469-CNTNAP2/tissue

**Table 3:** Normal human T cells express all the Neurotransmitters receptors which are the antigens of autoimmune antibodies present in a variety of neurological and psychiatric diseases.

Autoimmune antibodies against neurotransmitters receptors	Diseases in which some patients have anti-neurotransmitter receptor autoimmune antibodies	Do normal human T cells express the neurotransmitter receptors targeted by the autoimmune antibodies? Answer and few supporting Refs	Are human T cells damaged by autoimmune antibodies that target the neurotransmitter receptors that T cells express
**Glutamate receptor antibodies**			
AMPA-GluR3 antibodies	Epilepsy of several types, ‘Autoimmune Epilepsy’	YES, T cells express Glutamate AMPA GluR3 ionotropic receptors (78, 79, 80, 10, 102, 116, 100)	YES ! Normal human T cells are bound and then killed in vitro by epilepsy patient’s affinity-purified GluR3 **B** peptide antibodies (10) The patient’s GluR3 **B** antibodies on their own bind, induce production of Reactive Oxygen Species (ROS) and kill the normal human T cells in vitro, within 1 hr. only (10).
NMDA-NR1 antibodies	NMDA Encephalitis, Limbic Encephalitis, Herpes Simplex Virus Encephalitis, Slowly progressive cognitive impairment? Other?	YES, T cells express Glutamate NMDA ionotropic receptors that contain the NR1 and NR2 subunits (117, 173–176, 10)	Unknown.
			Not tested yet. Yet, interestingly, patients with NMDAR encephalitis and NMDA-NR1 antibodies were found to have lower frequencies of CD154-expressing NR1-reactive helper T cells than healthy controls, and produced significantly less inflammatory cytokines (52).
NMDA-NR2 antibodies	Neuropsychiatric SLE , Paraneoplastic Encephalitis, Mania, Schizophrenia, Slowly progressive cognitive impairments? Other?		
mGluR1 antibodies	Paraneoplastic Cerebellar Ataxia, Other?	YES, T cells express Glutamate metabotropic receptors (113, 114, 177, 178)	Unknown
			Not tested yet
mGluR5 antibodies	Ophelia Syndrome		
**Dopamine receptor antibodies**	Psychosis, Movement disorders: Parkinsonism, Dystonia, Chorea	YES, T cells express all types of Dopamine receptors (107–112, 179–182)	Unknown
			Not tested yet
**GABA receptor antibodies**	Limbic Encephalitis	YES, T cells express GABA receptors (120, 121, 183–187)	Unknown
			Not tested yet
**Acetylcholine receptor antibodies**	Myastenia Gravis, Chronic fatigue syndrome	YES, T cells express Acetylcholine receptors (118, 119, 188)	Unknown
			Not tested yet
**Serotonin (5-HT) receptor antibodies**	Autism, Developmental disorders, Non-autistic epilepsy	YES, T cells express Serotonin receptors (122–125)	Unknown
			Not tested yet
**Adrenaline and Noradrenaline receptor antibodies, mainly β1-adrenergic receptor antibodies**	Autoimmune myocarditis, Chagas disease (* see below).. Cardiomyopathy, Idiopathic dilated cardiomyopathy. *Chagas disease is an endemic parasitic disease of Latin American countries, caused by infection with the flagellate protozoan, Trypanosoma cruzi.	YES, T cells express adrenoceptors (126–134)	Unknown

Normal human T cells express on their cell surface receptors for most if not all the main Neurotransmitters. These include: GluRs, GABA-Rs, Dopamine-Rs, Ach-Rs, Serotonin-Rs and adrenergic receptors, that are the antigens of autoimmune antibodies present in patients with a variety of neurological diseases. Therefore, we hypothesize that the T cells of some patients with these neurological/psychiatric diseases could be damaged by the respective autoimmune antibodies,, leading to multiple pathological consequences.

This hypothesis calls for clinical and scientific investigations. In support of our hypothesis, our recent findings show that a specific type of Glutamate receptor autoimmune antibodies: GluR3 **B** peptide antibodies, kill normal human T cells in vitro. Indeed, affinity-purified GluR3 **B** peptide antibodies of epileptic NS patients, bind, induce ROS in, and kill normal human T cells in vitro, within a single hour (10).

Importantly, some of these autoimmune antibodies have been shown to induce potent pathological effects on neural cells, both *in vitro* and *in vivo*; to induce or promote seizures directly or indirectly; and to be associated with various neurological impairments (see **Table 1**, and Parts 3 and 4 of this article).

Type 1: Autoimmune antibodies to Glutamate receptor AMPA type, subunit GluR3, peptide **B**, called **GluR3B peptide antibodies, or GluR3B antibodies** (2–7, 9, 10, 13–16, 19–21, 24, 25, 28, 31, 32, 49 **),** (Part 4, **Tables 1**, **2** and **Figure 1**).

Type 2: Autoimmune antibodies to Glutamate receptor NMDA type, NR1 subunit, called **NMDA-NR1 antibodies** (50–57, 146–149, 10, 31, 34, 14, 36, 37, 39, 47, 63, 88);

Type 3: Autoimmune antibodies to Glutamate receptor NMDA type, subunit NR2, called **NMDA-NR2 antibodies** (9, 10, 14, 31, 76, 89–93, 150–165);

Type 4: Autoimmune antibodies to GABA receptors, called **GABA-R antibodies** (57, 58, 60–62, 64–67, 169–170, 33, 43);

Type 5: Autoimmune antibodies to Glutamic-acid-decarboxylase (GAD)65, called **GAD-65 antibodies** (13, 18, 26, 27, 29, 35, 36, 39, 44–46, 64, 66, 76, 166, 168);

Type 6: Autoimmune antibodies to Voltage-gated potassium channels (VGKC), usually called **VGKC antibodies** (18, 26, 27, 29, 30, 34, 35, 36, 39, 41, 42, 56);

Type 7: Autoimmune antibodies to Leucine-rich glioma-inactivated 1 (LGI1), called **LGI1 antibodies** (18, 34, 35, 38, 39, 42, 47, 48, 52, 55, 57, 59, 70, 71, 76, 146, 169);

Type 8: Autoimmune antibodies to Contactin-associated protein-like 2 (CASPR2), called **CASPR2 antibodies** (18, 30, 34–37, 55, 59, 72, 73, 169);

Type 9: Autoimmune antibodies to Glycine receptor (GLYR), called **GLYR antibodies** (36, 69, 74);

Type 10: Autoimmune antibodies to Cardiolipin (CLP), called **CLP antibodies** (13, 68, 75);

Type 11: Autoimmune antibodies to Beta2-glycoprotein-I (GP1), called **β2 GP1 antibodies** (13, 68, 75);

Type 12: Autoimmune antibodies to double-stranded DNA (dsDNA), called **dsDNA antibodies**, and to other nuclear proteins (13, 14, 16, 23, 158).

## 3. Several Autoimmune Antibodies Present in Epilepsy Patients Have Been Shown to Induce Pathogenic Effects *In Vitro*, and in Animal Models *In Vivo*

Several autoimmune antibodies that have been detected in the serum and CSF of epilepsy patients have demonstrated potent pathogenic activity *in vitro* and *in vivo*, which can harm the normal central nervous system (CNS) signaling and function, and induce or contribute to seizures directly or indirectly (2–6, 7, 9, 10, 16, 19, 25, 31–34, 49, 51, 54, 67, 70, 72–74, 89, 92, 147–153, 158, 160, 162, 163, 166–170).

The multiple evidences for the pathogenic activity of these autoimmune antibodies are summarized in **Table 1** which also contains citations of the articles that show each effect, and in **Figure 1**.

Additionally, Part 5 will elaborate only on GluR3 **B** peptide antibodies, due to their multiple unique features, potent detrimental effects, and recent discoveries explained therein. Part 5 will also review the current knowledge about the expression and function of the corresponding GluR3 subunit of Glutamate receptors of the AMPA type, which is the autoantigen of these autoimmune antibodies.

A few studies have reported large seizure burden with differing seizure semiology, and a high risk to develop status epilepticus or epilepsia partialis continua, among individuals with epilepsy and autoimmune antibodies [see for example (36)].

**Figure 2:**
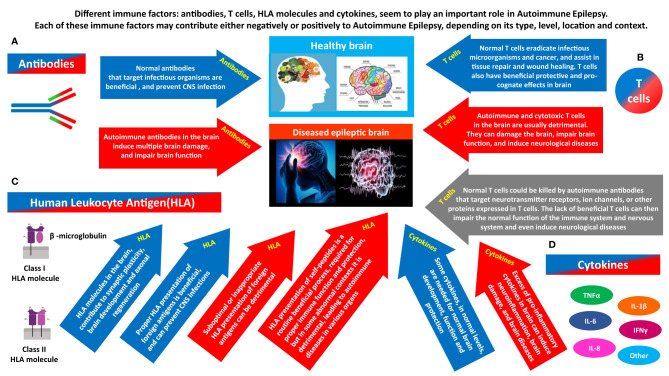
The different immune factors that play a role in Autoimmune Epilepsy, and the potentially opposing contribution of each. Different immune factors: antibodies, T cells, HLA molecules and cytokines, seem to play a major role in Autoimmune Epilepsy. Each of them can have a ‘double face’, and can contribute negatively or positively, depending on its type, level, timing, location and general context. **(A)** Antibodies can be beneficial or detrimental. Normal antibodies that target infectious organisms are beneficial, and prevent CNS infections. In contrast, if certain autoimmune antibodies are present in brain (whether due to their local Intrathecal production, or due to penetration into the brain from the periphery), they can damage, impair the activity, and even kill neural cells. By doing so, the autoimmune antibodies can cause multiple brain damages, and impair the general brain function, and even the function of many other organs that are dependent on normal brain activity for their own function. **(B)** T cells can be beneficial or detrimental. Normal T cells eradicate infectious microorganisms and cancer, and assist in tissue repair and wound healing. T cells also have beneficial protective and pro-cognate effects in brain. In contrast, Autoimmune and cytotoxic T cells in the brain are usually detrimental. They can damage the brain, impair brain function, and induce neurological diseases. On top of all that, another hypothesized pathway linking T cells and brain pathology, including epilepsy is the following: normal T cells could be killed by autoimmune antibodies that target Neurotransmitter receptors, ion channels, or other proteins expressed in T cells. The lack of essential T cells in the periphery and brain may then impair normal brain function, and even induce neurological diseases. **(C)** HLA can be beneficial or detrimental. HLA molecules in the brain, contribute to synaptic plasticity, brain development and axonal regeneration. In addition, proper HLA presentation of foreign antigens is beneficial, and can prevent CNS infections. In contrast, suboptimal or inappropriate HLA presentation of foreign antigens can be detrimental. In addition, with regards to HLA presentation of self-peptides: HLA presentation of self-peptides is a routine beneficial process, required for proper ongoing immune function and protection, but in some abnormal contexts it can be detrimental and lead to autoimmune diseases. **(D)** Cytokines can be beneficial or detrimental. Some cytokines, in normal levels, are needed for normal brain development, function and protection. In contrast, excess of pro-inflammatory cytokines in brain can induce neuroinflammation, brain damage, and even neurological diseases.

Based on the evidences summarized in the text of this paper, **Table 1** and **Figure 1**, and on the corresponding original cited papers, we envision that any autoimmune antibody present in a given epilepsy patient that can directly or indirectly impair the levels, signaling and function of Glutamate or GABA, or of their receptors, enzymes, ion channels, transporters or other associated proteins, (**Figure 2**), can in principle be detrimental and induce or promote seizures. Further, we foresee that any autoimmune antibody that can bind, impair and/or kill neural cells (**Table 1**) can induce seizures and additional neuropathology by various different mechanisms of action. Examples of optional autoimmune-mediated mechanisms of action are: activation, inhibition, degradation, internalization or modification of receptors, modulation of gating of ion channels, and of the related inward and outward ion currents, alteration of enzymes and signaling molecules, and others (**Figure 1**).

## 4. The GluR3 Subunit of Glutamate/AMPA Receptors Regulates Seizures, Breathing, and Sleep

Autoimmune GluR3 antibodies, and especially GluR3 **B** peptide antibodies, are found in a significant number of patients with enigmatic and uncontrollable epilepsy, have unique features, and induce multiple pathological effects *in vitro* and *in vivo* (2–7, 10, 13–15, 19–21, 23–25, 28, 31, 32, 49, 82). These effects are summarized in **Table 1**, and discussed below in the different sections of Part 4.

### 4.1 Expression and Localization of the AMPA GluR3 Subunit

The AMPA GluR3 subunit is expressed in the brain, mainly in the thalamus and cortex, and in the brainstem and spinal cord. It is located at centers responsible for respiratory control, cardiac control, and motor coordination (77). Additionally, normal human T cells, and also autoimmune T cells, express high levels of GluR3 on their cell surface, which is identical in sequence to the brain’s GluR3 (78, 79, 80, 10).

Within the brain, GluR3 immunoreactivity delineates a subpopulation of parvalbumin-containing interneurons in the hippocampus (81, 148).

GluR3 immunoreactivity was detected in all pyramidal neurons and astrocytes, and in most interneurons. GluR3 immunofluorescence (but not GluR1 or GluR2), is significantly elevated in somata of parvalbumin-containing interneurons, compared to pyramidal somata. GluR3 immunoreactivity is enriched in parvalbumin-containing perikarya at cytoplasmic and postsynaptic sites (81). Parvalbumin-containing interneurons are known as potent inhibitors of cortical pyramidal neurons, and are vulnerable in the brains of epilepsy patients. These findings suggest that the somata of these interneurons are enriched in GluR3, which may render them vulnerable to pathological states, and primarily Autoimmune Epilepsy, in which pathological GluR3 antibodies are present (Part 4.3, **Table 1**).

### 4.2 The GluR3 Subunit Regulates Seizures, Breathing, and Sleep

GluR3(-/-) knockout mice virtually lack electroencephalogram (EEG) signatures of non-rapid eye movement (NREM) sleep, as demonstrated by reduced EEG power in low-frequency bands (delta1, delta2 and theta) (77). Moreover, three of nine studied GluR3(-/-) knockout mice expressed seizure activity during wakefulness and sleep. These findings indicate that the deletion of the GluR3 gene may predispose to seizures. The GluR3 gene knockout also produced state-dependent respiratory modulation, with selective reduction in breathing rate during behavioral inactivity. Thus, the GluR3 subunits of ionotropic glutamate receptor of the AMPA type, seem to have diverse neurophysiological impact, modulating oscillatory networks for sleep, breathing and seizure generation (77).

### 4.3 The Autoimmune GluR3B Peptide Antibodies

Pathological GluR3 **B** peptide autoimmune antibodies (2–7, 10, 13–15, 19–21, 23–25, 28, 31, 32, 49, 82), **Table 1**, **Figure 1**), are a specific type of glutamate receptor autoimmune antibodies, which until now have only been detected in epilepsy patients.

The GluR3 **B** peptide autoimmune antibodies are directed against extracellular 24 amino acids (aa) GluR3 **B** peptide: NEYERFVPFSDQQISNDSSSSENR, corresponding to extracellular aa 372-395 of the GluR3 subunit of Glutamate AMPA receptors. This extracellular ‘**B**’ peptide has a unique agonist binding site within this ionotropic glutamate receptor, different from Glutamate’s own binding site (3, 82, 49, 6). The GluR3 **B** peptide antibodies bind the GluR3 **B** peptide, and subsequently can activate the AMPA receptor, kill neural cells by 3 mechanism: excitotoxicity, oxidative stress, and fixation of complement, induce severe brain damage of multiple types, induce or at least promote epilepsy, and induce behavioral and cognitive impairments (1, 2, 3, 5–7, 9, 10, 13, 15, 16, 19, 25, 31, 32, 49). The characteristics and activity of the GluR3 **B** peptide antibodies, are summarized according to 14 topics, in the below parts 4.3.1. - 4.3.14, and also in **Table 1** and **Figure 1**.

#### 4.3.1 GluR3 Antibodies Are Abundant in Intractable Epilepsy

Elevated levels of GluR3 antibodies, especially GluR3 **B** peptide antibodies, were found so far in serum of about 27% of >300 of persons with severe, intractable and enigmatic epilepsy of various types [(2–5, 10, 13–16, 20, 21, 23, 24, 28, and see a partial summary in a review article entitled: "Glutamate receptor antibodies in neurological diseases" (31)]. Recently, GluR3 **B** peptide antibodies were also detected in 86% of young individuals with the devastating and often fatal pediatric epilepsy: Nodding Syndrome (NS), which turns out to be an Autoimmune Epilepsy (10). GluR3 **B** peptide antibodies were also found in the CSF of some epilepsy patients [see for example (13, 23)].

#### 4.3.2 GluR3 Antibodies Appear So Far To Be Exclusive to Epilepsy

So far, the GluR3 antibodies, primarily the GluR3 **B** peptide antibodies, have only been detected in epilepsy patients. This contrasts with all the other autoimmune antibodies that have been found in persons with epilepsy, which are specified above in Part 2, and in **Table 1** and **Figure 1**; for partial summary, see the review article (31), and a recent article (10). Yet, we speculate that in the future, autoimmune GluR3 **B** peptide antibodies may also be found in persons with other autoimmune neurological diseases that share some features with Autoimmune Epilepsy.

#### 4.3.3 The GluR3B Peptide Antibodies Bind Neural Cells And Activate Glutamate AMPA Receptors

GluR3, and GluR3 **B** peptide antibodies, whose origin in either epilepsy patients, mice, rats, or GluR3 **B** peptide monolclonal antibody (mAb), bind neural cells (2, 3, 6, 7, 10, 25, 80, 82). Furthermore, the GluR3 and GluR3 **B** peptide antibodies have a very unique property, and that is that they are activating antibodies (3, 82, 49, 6). Affinity-purified GluR3 **B** peptide autoimmune antibodies activate by themselves ionotropic Glutamate/AMPA receptors that contain the GluR3 subunit, and induce the characteristic ion currents (3, 6, 49, 82).

Interestingly, we discovered that the GluR3 **B** peptide antibodies activate on their own both homomeric GluR3(o) receptors, and heteromeric AMPA receptor channels, which are composed of GluR3(o)/GluR2(o) or GluR3(o)/GluR2(i), without requiring any other neuronal, glial or blood ancillary molecules (49).

The affinity-purified GluR3 **B** peptide antibodies neither synergize nor interfere with Glutamate-evoked currents. They act like partial Glutamate/AMPA agonists, but, interestingly, activate AMPA receptors *via* their own corresponding ‘**B** peptide’ binding site (49). The binding of the affinity-purified GluR3 **B** peptide antibodies to homomeric GluR3 is reversible and of low affinity, and does not alter their subsequent response to an AMPA receptor agonist (3, 82, 49).

#### 4.3.4 GluR3B Peptide Antibodies Induce Excitotoxicity

Excitotoxicity is neuronal death process, induced by massive release of Glutamate to the extracellular space, after lysis of neurons (83, 84). Glutamate-induced excitotoxicity causes severe brain damage in numerous neurological diseases, injuries and traumas, including epilepsy (83, 84).

The brain damage caused by excess Glutamate *via* excitotoxicity, spreads very rapidly from the primary focus into neighboring loci, destroying more and more brain regions, alike a tsunami storm in the brain.

Interestingly, the GluR3 **B** peptide antibodies kill on their own neural cells growing in tissue culture by inducing excitotoxicity. i.e. activation of the AMPA receptor's ion channel (6). Both the activation of the GluRs and the neuronal death induced by GluR3 **B** peptide antibodies were blocked by CNQX, a selective AMPA receptor antagonist. The killing was independent of complement. This indicates a mechanism of excitotoxicity - neuronal death due to over-activation of receptors, alike caused by excess of Glutamate. The excitotoxic neuronal death induced by GluR3 **B** antibodies took place primarily via apoptosis. The neuronal killing capacity of purified GluR3 **B** peptide antiboodies was completely and specifically blocked by preincubation with the GluR3 **B** peptide (6).

#### 4.3.5 GluR3B Peptide Antibodies Induce Reactive Oxygen Species (ROS) in Human Neural Cells and Kill These Cells

Affinity-purified GluR3 **B** peptide antibodies of NS patients were recently shown to induce *in vitro* rapid and robust production of pathological ROS in human neural cells, within a single hour (faster times were not tested), and to kill these neural cells (10).

#### 4.3.6 GluR3B Peptide Antibodies Induce ROS in Normal Human T Cells and Kill These cells

Affinity-purified GluR3 **B** peptide antibodies of NS patients were recently shown to induce *in vitro* rapid production of ROS in normal human T cells that express high levels of GluR3, and to kill these T cells within a single hour (10). Thus, epilepsy patient's GluR3 **B** peptide antibodies cause in vitro ROS production in both human neural cells and naive T cells. and kill both cell types (10).

#### 4.3.7 GluR3 Antibodies Can Kill Astrocytes and Neurons by Complement Fixation

GluR3 antibodies raised in rabbits (not patient's GluR3 **B** peptide antibodies) were shown to destroy cultured cortical cells in a complement-dependent manner (5, 19). In a mixed primary neuronal–glial cultures of rat cortex. astrocytes were unexpectedly found to be the principal target of the cytotoxic effects, while neurons were destroyed to a lesser extent. Astrocyte vulnerability was rescued by transfection with complement regulatory proteins, and neuronal resistance was defeated by impairing complement regulatory protein function (5, 19). 

#### 4.3.8 The Major Histocompatibility Complex (MHC) Influences the Magnitude of GluR3B Peptide Antibodies Production in Mice

The immunogenetic background, and the MHC specifically, influences the level of, and/or susceptibility to, the production of autoimmune GluR3 **B** peptide antibodies (7). This conclusion is based on the discovery that BALB/c (H-2d), C3H/HeJ (H-2k), SJL/J (H-2s) and DBA (H-2d) developed high titers of specific GluR3 **B** peptide antibodies following immunization with the GluR3**B** peptide, but C57BL/6 (H-2b) mice did not. The hierarchy of the GluR3 **B** peptide antibody levels in these different mice strains was: C3H/HeJ and DBA/J > BALB/c > SJL/J/6 (H-2b) >>>>> C57BL/6 (7, 32).

#### 4.3.9 Anti-GluR3B Peptide T Cells Are Being Produced in Mice, in Addition to Anti-GluR3B Peptide Antibodies, Following Immunization With the GluR3B Peptide

Mice immunized with the GluR3 **B** peptide, developed GluR3 **B** peptide-specific T cells, in addition to GluR3 **B** peptide-specific antibodies (7). Furthermore, the splenocytes of the GluR3 **B**-immunized mice expressed significantly biased frequencies of particular TCR Vβ families: Vbeta11, Vbeta7 and Vbeta8 (7). This skewed pattern of expression of particular TCR-Vβ families suggest that a clonal proliferation of specific autoimmune T cell populations took place in the spleens of mice, in response to the immunization with the GluR3 **B** peptide.

Based on these findings in mice, we hypothesize that patients with Autoimmune Epilepsy contain in their body both autoimmune GluR3 **B** peptide antibodies, and autoimmune GluR3 **B** peptide T cells, and that both can damage the brain and additional organs and tissues which contain GluR3-expressing cells.

#### 4.3.10 GluR3B Peptide Antibodies Induce Severe Brain Damage *In Vivo*

GluR3 **B** peptide antibodies produced in mice (7, 10, 32), rats (25) or rabbits (2, 5), as well as epilepsy patient’s IgG’s rich in such GluR3 **B** peptide antibodies, were shown to induce severe brain damage of multiple types in animal models (2, 5, 7, 10, 19, 25 , 32). See also (31) for review, and **Table 1** for summary).

In one study, normal mice that developed specific GluR3 **B** peptide antibodies following immunization with the GluR3 **B** peptide exhibited multiple brain pathology (7). The GluR3 **B**-immunized mice expressed: 1. Thickening of the cerebral meninges, 2. Perivascular lympho-mononuclear cell infiltration; 3. Occasional pathologic gliosis in the cerebrum; 4. Cerebellar cortical abiotrophy, with loss of neurons, from both the Purkinje and granule cell layers; 5. Moderate to severe spongiform degeneration, mainly in the cortex, in the cerebellar white matter, and in few foci in the cerebrum and spinal cord (7).

In another study, normal rats that developed specific GluR3 **B** peptide antibodies following immunization with the GluR3 **B** peptide exhibited multiple brain pathologies, among them fewer NeuN^+^ mature neurons in the superficial layer VI in both the motor cortex and the somatosensory cortex (25). This neuropathology was accompanied by the appearance of more DCT^+^ positive newly born immature neurons in the subventricular zone. The GluR3 **B**-immunized rats also had almost twice the GFAP^+^ astrocytes in both the motor cortex and the somatosensory cortex. The GFAP^+^ astrocytes had the characteristic reactive appearance: hypertrophy of their cellular processes, filled with increased amounts of GFAP, indicative of reactive gliosis (25).

Furthermore, in our recent study (10) we discovered that when NS patient’s purified IgG, which contained elevated levels of GluR3 **B** peptide antibodies, was released continuously in brains of normal naive mice for 1 week (via a mini pump), the patient's purified IgG induced seizures (see Part 4.3.11) and also: 1. Cerebellar Purkinje cell loss, 2. Degeneration in the hippocampus and cerebral cortex, 3. Elevation of CD3^+^ T cells, and of activated Mac-2^+^ microglia and GFAP^+^ astrocytes, in both the gray and white matter of the cerebral cortex, hippocampus, Corpus callosum and cerebellum (10).

#### 4.3.11 Purified IgGs of Epilepsy Patients, Rich in GluR3B Peptide Autoimmune Antibodies, Induce Seizures and Profound Brain Pathology in Normal Mice

We continuously released in brains of normal mice (24/7 for 1 week, by mini-pump) NS patient's purified IgGs containing high levels of GluR3 **B** peptide antibodies. Then we measured the electrical activity in the brain for several weeks, by continuous video-EEG monitoring. We revealed that the patient’s purified IgG induced seizures (10). All 4 mice (100%) receiving NS patient's purified IgG developed seizures, compared to only 2 of 5 (40%) of mice receiving healthy subject's IgG (10). Total of 40 seizures events occurred in mice receiving NS patient's IgG, compared to only 11 in mice receiving healthy subject's IgG. The mean number of seizures per day in mice receiving NS patient's IgG was 0.71, compared to 0.17 in mice receiving healthy subject's IgG. All these effects were statistically significant. Taken together, the results of these in vivo experiments show that the NS patient's IgG, which is rich in GluR3 **B** peptide antibodies, can by itself induce seizures in normal mice, and that such seizures are significantly more frequent than nonspecific seizures induced by healthy human IgG. Yet, as expected, the artificial continuous release of healthy human IgG in the brain of mice for 1 week also induced some non-specific seizures (10). We currently continue these *in vivo* video EEG studies, and testing IgGs of other epilepsy patients (not NS patients), in which we find strong indications for Autoimmune Epilepsy.

#### 4.3.12 GluR3B Peptide Antibodies Reduce the Seizure Threshold *In Vivo* In Normal Mice

Normal DBA/J mice that developed GluR3 **B** peptide antibodies following immunization with the GluR3 **B** peptide, exhibited reduced threshold to PTZ-induced seizures (32). In the few control groups of mice not having GluR3 **B** peptide antibodies, the PTZ-induced seizure severity scores, and the percentages of animals developing generalized seizures declined in response to decreasing PTZ doses. In contrast, both parameters remained unchanged/high in the GluR3 **B** antibody positive mice, showing that these mice were more susceptible to seizures. The seizure scores associated significantly with the levels of the GluR3 **B** antibodies. Interestingly, the GluR3 **B** antibody positive mice expressed significant impairments in 3 behavioral tests, which are specified in (32), and in the below Part 4.3.13.

#### 4.3.13 GluR3B Peptide Antibodies Induce Behavioral and Motor Abnormalities *In Vivo* in Animal Models

Mice that developed GluR3 **B** peptide antibodies following immunization with the GluR3 **B** peptide, exhibited significant abnormalities in three behavioral and/or motor tests: they were significantly more anxious in Open-Field test, fell faster in the RotaRod test, and fell more in the Grip test, compared to the control mice (32). Thus, GluR3 **B** peptide antibodies can induce on their own, in animal models, behavioral and/or motor impairments, in addition to epileptic seizures.

#### 4.3.14 GluR3B Peptide Antibodies Associate With Some Cognitive, Psychiatric, and/or Behavioral Abnormalities In Epilepsy Patients

In a study on 41 young patients with severe, intractable and enigmatic epilepsy, we detected elevated levels of GluR3 **B** peptide antibodies in 21 of them (>50%). Of these 21 patients, 19 patients (90%) had learning problems, 16 (76%) attention problems, and 15 (71%) psychiatric problems (15).

In contrast, among the 20 patients with severe, intractable and enigmatic epilepsy in which we did **not** detect GluR3 **B** peptide antibodies, only 6 (30%) had learning problems (p < 0.0001), 5 (25%) attention problems (p = 0.0017), and 2 (10%) psychiatric problems (p < 0.0001) (15). Two interpretations of these findings are possible. The first interpretation is that neurobehavioral abnormalities occur more frequently in epilepsy patients that have elevated levels of GluR3 **B** peptide autoimmune antibodies, and these GluR3 **B** peptide autoimmune antibodies may even be the direct cause of such neurobehavioral impairments.

The second interpretation is that GluR3 **B** peptide autoimmune antibodies are more abundant in epilepsy patients already having neurobehavioral abnormalities.

Based on all the findings summarized in Part 4, **Table 1** and **Figure 1**, and described in the corresponding cited papers, we strongly recommend testing for GluR3 **B** peptide autoimmune antibodies, and preferably also for GluR3 **B** peptide T cells, in all individuals with intractable epilepsy, because these autoimmune antibodies can induce multiple pathological effects, via several mechanisms of action.

## 5. The ‘Fateful Duel’ That Triggers Epilepsy, Between the Two Titanic Neurotransmitters: Excitatory Glutamate vs. Inhibitory GABA, Spreads to the ‘Autoimmunity Battlefield’ When Their Receptors, Enzymes or Other Associated Proteins, Are Targeted by Autoimmune Antibodies

Normal brain signaling and function requires tightly regulated and carefully-maintained balance between electrical and biological excitation and inhibition. Imbalance between excitation and inhibition, and especially excess excitation, can induce epileptic seizures.

Glutamate is the main excitatory neurotransmitter, while GABA is the main inhibitory neurotransmitter in neural cells of the nervous system. Glutamate is the metabolic precursor of GABA, which can be recycled through the tricarboxylic acid cycle to synthesize Glutamate. **Figure 1** shows the biosynthetic pathway of Glutamate and GABA, and all the enzymes involved. **Figure 1** also schematically shows the crucial balance between Glutamate-induced excitation and GABA-induced inhibition.

Changes in the production, release, levels, receptors, signaling, function, uptake, degradation and internalization of either Glutamate or GABA, or of their receptors, can influence cortical excitability. And any type of over excitation can eventually lead to seizures (85). Excess of Glutamate or of Glutamate receptor agonists NMDA and AMPA was shown to lead to seizures in animal models.

Epilepsies are known to result from long-lasting plastic changes in the brain, affecting neurotransmitter release, the properties of receptors and channels, synaptic reorganization and astrocyte activity. There is considerable evidence for alterations in glutamatergic and GABAergic synaptic transmission in the origin of the paroxysmal depolarization shifts that initiate epileptic activity (86, 87, 85).

**Figure 1** is based on a large number of different articles we have reviewed, especially in order to shed light on the topic of autoimmunity-mediated impairments in Glutamate and GABA signaling in Autoimmune Epilepsy. **Figure 1** shows the autoimmune antibodies found in subpopulations of epilepsy patients, that can directly or indirectly impair the level and/or function of Glutamate and GABA, or of their receptors, enzymes and other associated proteins.

We envision that by affecting the ‘Glutamate *vs*. GABA duel’, each of these autoimmune antibodies can impair normal brain signaling and function, trigger epileptic seizures, and induce or intensify severe brain damage.

We further suspect that the autoimmune antibodies listed in **Figure 1**, which are present in significant number of patients with intractable epilepsy, can also induce a broad spectrum of functional impairments in peripheral organs in which Glutamate and GABA and their receptors play an important role.

## 6. Specific Autoimmune Antibodies Correlate With Cognitive, Behavioral and Psychiatric Impairments In Epilepsy Patients. Glutamate Receptor Autoimmune Antibodies Induce by Themselves Cognitive and Behavior Abnormalities in Mice

Among epilepsy patients, those with certain types of autoimmune antibodies have been shown to have more neurobehavioral and cognitive comorbidities (15, 35, 39, 45, 50, 53, 56, 63, 90, 91, 92, 93, 150, 151). This was revealed in regard to patients with any of three types of glutamate receptor antibodies: AMPA-GluR3 **B** peptide antibodies (15), NMDA-NR1 antibodies (35, 50, 53, 56, 63, 88), or NMDA-NR2 antibodies (89, 90, 91, 92, 93, 150, 151), and for antibodies directed against TPO, GAD-65, VGKC and/or LGI1 (39, 45).

An example of a study that showed such correlation is the one that was performed on 112 epilepsy patients with unknown etiology, and that detected autoimmune antibodies in 39 (34.8%) patients: 15 (13.4%) had TPO antibodies, 14 (12.5%) had GAD65 antibodies, 12 (10.7%) had VGKCc antibodies (4 of whom were positive for LGI1] antibodies), and 4 (3.6%) had NMDA-R antibodies (39).

More than one antibody was detected in 7 patients (6.3%): 3 (2.7%) had TPO antibodies and VGKCc antibodies, 2 (1.8%) had GAD65 antibodies and VGKCc antibodies, 1 had TPO antibodies and GAD65 antibodies, and 1 had Hu antibodies and GAD65 antibodies. Thirty-two patients (28.6%) had a single type of antibodies (39). Unfortunately, the patients were not tested in this study for GluR3 **B** peptide antibodies.

Certain clinical features, such as autonomic dysfunction, neuropsychiatric changes, viral prodrome, faciobrachial dystonic spells or facial dyskinesias, and mesial temporal sclerosis abnormality on magnetic resonance imaging, correlated with seropositivity (39).

In another study, we found that among 71 consecutive pediatric patients with severe intractable epilepsy (20 with generalized epilepsy, 51 with partial epilepsy), neurobehavioral abnormalities occurred significantly more frequently in those with GluR3 **B** peptide antibodies (15) (see Part 4.3.14 for a summary of these findings).

We suggest two possible explanations for all these findings. For one, certain types of autoimmune antibodies, among them GluR3 **B** peptide antibodies and others, may induce or promote neurobehavioral abnormalities in epilepsy patients. Alternatively, patients suffering from epilepsy and other and neurobehavioral abnormalities could be more susceptible to the production of certain types of autoimmune antibodies, among them GluR3 **B** peptide antibodies and others. The human data do not provide validation for either scenario, but both possibilities can lead to severe neurological problems in patients.

Only a few *in vivo* studies showed, in animal models, a correlation between specific types of autoimmune antibodies that are present in individuals with epilepsy, and cognitive or behavioral abnormalities (**Table 1**). In one study, already described above in Part 4.3.13, mice that developed high levels of GluR3 **B** peptide antibodies following immunization with the GluR3 **B** peptide were significantly more anxious in the Open-Field test, fell faster in the RotaRod test, and fell more in Grip test, compared to few groups of control mice (32).

In other studies, mice developed NMDA-NR2 peptide (aa 283-287)/dsDNA cross-reactive antibodies following immunization (150, 151, 153, 163). Then, the blood-brain barrier (BBB) was compromised, for allowing these autoimmune antibodies to gain access to the brain. These mice expressed neuronal death, cognitive dysfunction and emotional disturbance (150, 151, 153, 163). Interestingly, the manner by which the BBB was disrupted determined the affected brain region, and the type of CNS impairment: Lipopolysaccharide led to antibody-mediated damage to the hippocampus and memory disturbance, while Epinephrine led to neuronal loss in the amygdala and behavioral change marked by aberrant Pavlovian fear conditioning (150, 151, 153, 163).

Taken together, these finding, and others not cited herein, demonstrate that certain types of autoimmune antibodies, primarily three types of Glutamate receptor autoimmune antibodies, directed against either: AMPA-GluR3, NMDA-NR1 and NMDA-NR2, can induce cognitive and behavior impairments in mice. We hypothesize that they can do so in humans as well.

## 7. Most Autoantigens in Autoimmune Epilepsy Have Very Dynamic Character and Activity. These Features May Increase Their Antigenicity, And The Severity Of The Damage Caused By The Autoimmune Antibodies That Target Them 

Most of the self-antigens targeted by autoimmune antibodies present in epilepsy patients (**Table 1**, **Figure 1**, Part 2) are very dynamic proteins. These include Neurotransmitter receptors, ion channels, key enzymes and/or their associated membranal proteins. Most if not of them undergo frequent rapid changes throughout their lifetime, such as: conformational changes, ligand-induced changes, voltage-mediated gating, lateral movements and polarization on the membrane, clustering, cleavage, uptake, internalization, re-cycling, turnover, physical and functional interactions with other proteins, and others.

We hypothesize that the ever changing ‘frenetic’ nature and activity of these self-proteins ‘mislead’ the immune system and increases the probability that these proteins are inadvertently viewed in certain contexts as foreign antigens, leading to the production of detrimental autoimmune antibodies against them.

We further speculate that the very important, active, constantly changing, and tightly regulated activity and turnover of these proteins, is also responsible for the very severe neuropathological effects and consequences induced by autoimmune antibodies that target them.

## 8. The Membranal and Synaptic Localization of Most of the Autoantigens May Increase Their Predisposition to Autoimmunity, and the Severity of the Pathological Effects and Consequences Induced by Autoimmune Antibodies That Target Them

Most self-proteins that become autoantigens of detrimental autoimmune antibodies in subpopulations of epilepsy patients, are expressed on the plasma membrane, and contain extracellular domains that harbor critical binding sites and/or activation domains within, while some other self-proteins are secreted (**Table 1**).

We suspect that the synaptic localization and extracellular binding site of the self-proteins increases their antigenicity, and the risk of pathogenic autoimmunity against them, due to several reasons. First, the extracellular domains of these proteins, which undergo frequent dynamic changes (among then those mentioned above in Part 7), are most probably constantly ‘sensed’ by patrolling and surveilling immune cells.

Second, the respective extracellular domains are often very sensitive to various changes that occur in the extracellular milieu in various physiological and pathological contexts, and respond rapidly.

Third, we envision that once autoimmune antibodies against these autoantigens are produced, their synaptic localization increases both the easiness and rapidness by which they are accessed, bound and impaired by the respective autoimmune antibodies, and subsequently increases the severity of the pathogenic autoimmune consequences.

## 9. The mRNAs and/or Proteins of the Autoantigens Are Expressed in Many Peripheral Tissues That Could Be Damaged By Autoimmune Antibodies, in Addition to the Brain

We have raised a new hypothesis, that on top of the autoimmune-mediated brain damage in Autoimmune Epilepsy, the function of some peripheral organs of epilepsy patients could also impaired by the harmful activity of the patient’s autoimmune antibodies, because the antigens are also expressed in these peripheral organs, and not only in the brain. For testing this hypothesis, we searched for information on the expression of the gene, mRNA and protein of each of the self-proteins/autoantigens in the various body tissues.

**Table 2** shows the gene name, main protein function/s, and tissue distribution of the mRNA and protein of seven major self-proteins targeted by key autoimmune antibodies in subpopulations of epilepsy patients.

This table is based on two very reliable databases: Uniprot - www.uniprot.org, and Gene Card - www.genecards.org.

The data shown in **Table 2** reveals two worrying facts.

First, the mRNA of each self-protein/antigen is widely expressed in variable levels in many tissues and organs throw-out the body, in addition to the brain!

Second, in many of these organs, the protein expression of the corresponding self-protein/antigen was never tested!

As a reminder, the default of mRNAs is rapid and efficient translation into proteins, as currently evident for example by the SARS-Cov-2 mRNA vaccines. The mRNA of the virus contained within these vaccines is very quickly translated into a protein in the body of the vaccinated people. Then, the viral protein triggers the production of antibodies against it, which are the ones that protect the vaccinated people.

We therefore envision that in principle, the mRNAs of the self-proteins relevant to Autoimmune Epilepsy could also be translated into proteins in peripheral organs and tissues. If this does occur, it is logical to assume that the peripheral organs and tissues need these self-proteins for their normal activity, and that if these are attacked by autoimmune antibodies, the proper functioning of these peripheral organs will be impaired, in addition to the impaired brain function.

To the best of our knowledge, this is the first time there is a recognition of the biological fact that the mRNAs of the self-proteins that become targets of autoimmune antibodies in Autoimmune Epilepsy are expressed in so many tissues throughout the body. In addition, this is probably the first time it is hypothesized that that the function of several peripheral organs of epilepsy patients could be impaired due to autoimmune-mediated damage caused by autoimmune antibodies that target antigens expressed both in the brain and in these peripheral organs.

Investigations of this topic, on a number of scientific and clinical levels, is very important.

## 10. Cause-Effect Relationships and Timing of Autoimmune Epilepsy

Autoimmune Epilepsy was first diagnosed and described in pioneering papers during 1994-2002, that is 28 years ago [(2–7, 20, 21, 1) - the cited papers are listed here in the chronological order of their publication]. The term "Autoimmune Epilepsy" was first coined in 2002 (1). Although 20 years past since then, Autoimmune Epilepsy is still not taken seriously into consideration by many neurologists and epileptologists. Some are still ignorant to Autoimmune Epilepsy, while others do not acknowledge this condition as a clinical entity in itself, and believe that the autoimmunity is only a secondary, late and non-specific result (epiphenomenon) of epileptic seizures, and hence a negligible phenomenon that does not require serious clinical consideration. Accordingly, such clinicians do **not** diagnose autoimmune antibodies and T cells in severe intractable epilepsy patients, and do **not** treat patients that actually suffer from Autoimmune Epilepsy with suitable anti-autoimmunity therapy. As a result of that, some patients keep suffering from Autoimmune Epilepsy that is not diagnosed and not treated properly.

In an attempt to overcome and refute the prevalent misconceptions, we will raise herein four points. We hope that this discussion will encourage new thoughts, understanding, and actual scientific and clinical acts.

**Point 1**: Most human clinical studies cannot reveal the timing of past events and cause-effect relationships. Only animal models can do so. By default, clinicians see patients too late - only after the symptoms have become clear and can be diagnosed. This is therefore retrospectively, days, weeks, months or even years after the primary trigger and events initiated the disease. Therefore, in patients it is often impossible to determine unequivocally what caused what and when. Accordingly, the claim that autoimmunity is necessarily epiphenomena is rootless.

**Point 2**. Autoimmune antibodies and/or elevated levels of immunoglobulins of various types, are detected in some intractable patients very early after the first diagnosis of epilepsy, indicating they are **not** epiphenomena of long-standing refractory seizures (29, 30, 94).

Indeed, in few studies elevated levels of immunoglobulins were revealed in some intractable epilepsy patients, when the epilepsy was first diagnosed. This clinical fact suggests that the autoimmune antibodies in these patients preceded the epilepsy, and that they were **not** a nonspecific late epiphenomena of long-lasting persistent refractory seizures (29, 30, 94).

**Point 3**: Only animal models can teach reliable cause-effect relationships, kinetics, scenario of events, and mechanisms of action.

A few animal models of Autoimmune Epilepsy, in normal mice, rats or rabbits, revealed *in vivo* that certain autoimmune antibodies can induce seizures, and/or reduce the seizure threshold, and also bind and kill neural cells in several brain regions, and damage the brain. Examples of such *in vivo* pathological effects of autoimmune antibodies in normal animals can be found in (2, 5, 6, 7, 10, 25, 32, 49, 51, 54, 70, 72, 73, 74, 92, 147, 149, 150, 151, 153, 163, 169, 170), **Table 1** and Part 4.3 above. These findings demonstrate that some pathological autoimmune antibodies can in fact induce or promote seizures and profound brain impairments in a healthy non epileptic body (**Table 1**). That said, in humans, is is logical to assume that some autoimmune antibodies may precede the epilepsy, while others may be produced after its outburst.

**Point 4:** We claim that for epilepsy patients themselves, the time and reason for the autoimmune outbreak does not really matter, since once detrimental autoimmune antibodies and/or T cells are present, they can cause massive damage, and continue to do so for years, regardless of the time and cause of their production.

Thus, it is very important to test patients for specific autoimmune antibodies and T cells as early as possible, and to repeat the diagnostic tests periodically in subsequent years.

If the autoimmune antibodies and/or T cells are found, all available strategies should be attempted to silence or remove them. See some optional therapeutic strategies in Part 14 and in (17, 18, 8, 38–43, 148, 11, 12, 45, 53, 57, 60, 61, 64, 69, 71, 76, 88, 146, 148).

Without such anti-autoimmunity treatment, the autoimmunity antibodies and T cells can persist for years, and cause chronic increasing damage.

## 11. Intermolecular Epitope Spreading in Autoimmune Epilepsy

Epitope spreading (95) is diversification of B cell and/or T cell responses over time, from the initial dominant epitope to secondary epitope/s.

Intramolecular epitope spreading consists of the diversification of the immune response in the same autoantigen. Intermolecular epitope spreading commonly involves a number of different antigens of a single macromolecular complex, or that co-localize in the same anatomical site.

In contrast to intramolecular and intermolecular epitope spreading, autoimmune reactivity that spreads from a single autoantigen to multiple antigens through **cross-reactivity** is **not** considered authentic epitope spreading (95).

In some studies on Autoimmune Epilepsy, different types of autoimmune antibodies, of those specified in Part 2 and in **Tables 1**, **3** and **Figure 1**, were found in individual patients, see for example (39, 13, 29, 30).

In a study, on 416 epilepsy patients, composed of two distinct cohorts of adults (aged 16 years and over) with either established epilepsy (n = 235) or new‐onset epilepsy (n = 181), a total of 46 epilepsy patients (the two cohorts combined) were found to have serum antibodies to one or more of the following antigens: VGKC, NMDA receptor, GAD, or Glycine receptor (29). Few patients had high levels of different autoimmune antibodies (29). GluR3 **B** peptide antibodies were unfortunately not tested in this study.

Interestingly, the prevalence of antibodies did not differ, individually or collectively, between patients with established and newly diagnosed epilepsy, or between patients with generalized and focal epilepsy (29).

In another study, on serum of 112 patients with new-onset epilepsy or established epilepsy of unknown etiology (39), autoimmune antibodies were found in 39 (34.8%) patients. Fifteen patients (13.4%) had TPO-antibodies, 14 (12.5%) had GAD65 antibodies, 12 (10.7%) had VGKC antibodies, 4 had LGI1 antibodies, and 4 (3.6%) had NMDA receptor antibodies. More than one type of autoimmune antibodies was detected in 7 patients (6.3%). Three patients (2.7%) had TPO-antibodies and VGKC antibodies, 2 had GAD65 antibodies and VGKC antibodies, 1 had TPO antibodies and GAD65 antibodies, and 1 had Hu antibodies and GAD65 antibodies (39). Specific GluR3 **B** peptide antibodies were unfortunately not tested in this study too.

In yet another study on 114 children (aged 2 months to 16 years) with new-onset seizures and 65 controls (30), eleven (9.7%) patients were found to be positive for one or more of the following antibodies: VGKC complex antibodies (n = 4), CASPR2 antibodies (n = 3), NMDA receptor antibodies (n = 2), or VGKC-complex and NMDA receptor (n = 2). None of the patients had antibodies to GAD, contactin-2, or to Glycine receptor, AMPA receptors or GABA receptors (30). Specific GluR3 **B** peptide antibodies were not tested in this study too.

In our recent paper (10), we revealed that epileptic NS patients have autoimmune antibodies to three different extracellular peptides of ionotropic glutamate receptors: 1. AMPA-GluR3 **B** peptide antibodies (found in 86% of tested patients), **2**. NMDA-NR1 peptide antibodies (77%), **3**. NMDA-NR2 peptide antibodies (87%).

In principle, we envision two possible scenarios for the production of these different GluR antibodies. For one, these GluR autoimmune antibodies could have been produced at the same time. Alternatively, they could have been produced sequentially, due to epitope spreading, involving different peptidergic autoantigens present within different GluR subunits expressed in the same cells. Interestingly, we did **not** find evidences for general nonspecific epitope spreading in these NS patients, since the patients did **not** have any of 26 other well-known autoantibodies that target the nervous system in different autoimmune neurological diseases (10).

In yet another earlier study, we detected in the serum of RE patients - an Autoimmune Epilepsy, elevated levels of GluR3 **B** peptide antibodies, and clinically elevated levels of antibodies to: GAD, Cardiolipin, β2GPI and the nuclear-antigens SS-A and RNP-70 (13).

Taken together, the evidences revealed in all the studies cited herein, and in several others, indicate that there is most probably intermolecular epitope spreading in Autoimmune Epilepsy, from the initial process that caused it, throughout the months and years that follow. Yet, it seems there is a great inter-individual variability with regard to the intermolecular epitope spreading between different patients with Autoimmune Epilepsy.

## 12. T Cells Have Different ‘Faces’ in The Brain and In Autoimmune Epilepsy

Different T cells seem to have different 'faces' and different contributions to the healthy brain and the diseased brain. And with regards to Autoimmune Epilepsy, we envision at least five different means of involvement of T cells, depending on their type and context (illustrated schematically in **Figure 2**, and summarized below).

Our hypothesis rests on very different evidences, which we combine and formulate here, for the purpose of this discussion.

First, normal beneficial T cells are needed for normal brain function (97, 98, 101, 96, 99), and have beneficial roles in various neurological diseases, ranging from tissue protection to regeneration [for review see (101), and for short summary see Part 12.1 below]. Thus, the presence of normal T cells in the brain is, or can be, in normal conditions, beneficial, **not** necessarily detrimental, as commonly assumed.

Second, we hypothesize that in cases where the primary cause of Autoimmune Epilepsy could be is an infectious organism, the recognition and eradication of the infectious organism by T cells could be critical for protecting the brain from the direct and indirect hazard effects of such infection. The effectiveness of the T cell response against the infectious organisms depends on several factors, the most important of which is probably the efficiency and level of the presentation of the infectious organism’s foreign antigens to T cells by the human HLA molecules (see Part 13).

Third, normal healthy T cells are killed *in vitro* by epilepsy patient’s autoimmune glutamate receptor antibodies: GluR3 **B** peptide antibodies, as we recently discovered (10). The GluR3 **B** peptide antibodies can kill normal human T cells, since the T cells express their target antigen: GluR3 on their cell surface (detected in several studies, by several methodologies) (78, 79, 80, 10, 116, 100) (see **Table 3** and Part 12.3), as do neural cells.

We envision that T cells may be damaged also in various other neurological diseases, by autoimmune antibodies to other Neurotransmitter receptors that they express (**Table 3** and Part 12.3).

Fourth, it is well known that autoimmune and/or cytotoxic T cells can undoubtedly induce profound brain pathology and even epilepsy (see Parts 12.4 and 12.5).

Fifth, T cells (that could be either beneficial or detrimental) could be recruited to the brain by autoimmune antibodies of epilepsy patients, as we found in a video EEG animal model of Autoimmune Epilepsy (10). This was evident by the observation that when purified IgG of epileptic NS patients rich in GluR3 **B** peptide antibodies was released continuously in brains of normal mice for 1 week, the IgG caused an elevation of CD3^+^ T cells, as well as activated microglia, in specific brain regions - both in the gray and white matter of the cerebral cortex, hippocampus, corpus calossum and cerebellum of the mice (10) (see also Part 12.6).

Three of these five different ‘T cell faces’ are discussed separately in further depth the below sections.

### 12.1 The Healthy Brain Needs Healthy T Cells

Normal healthy T cells are required for proper brain function (97, 98, 101, 96, 99). Indeed, T cells are needed for: cognition, spatial learning and memory, and adult neurogenesis (158–161). T cells are also needed in brain for neuroprotection, for decreasing secondary neuronal degeneration, for increasing neuronal survival and regeneration after CNS injury, and for limiting CNS inflammation and damage upon injury and infection (97, 98, 101, 96, 99). In addition to the beneficial roles of T cells in the healthy brain, their presence and activity in neurological diseases requires a different and updated approach, in light of the findings of recent years. This is because while the pathogenic effects and mechanisms of T cells in several CNS disorders are well-established, more recent studies have uncovered compelling beneficial roles of T cells in neurological diseases, ranging from tissue protection to regeneration (reviewed in 101). These beneficial effects are mediated primarily by helper CD4^+^ T cells.

In their recent paper, Evans et al review the beneficial impact of T cell subsets in a range of neuroinflammatory and neurodegenerative diseases including: Multiple Sclerosis, Alzheimer’s disease, Parkinson’s disease, Amyotrophic Lateral Sclerosis (ALS), stroke, and CNS trauma. Both T cell-secreted mediators and direct cell contact-dependent mechanisms deliver neuroprotective, neuroregenerative and immunomodulatory signals in these diseases (101). 

Based on all the above, we hypothesize that if normal T cells are either dying, exhausted, dysfunctional, impaired or even just very low in number, both the immune system and the nervous system will sense this T cell problem and be negatively affected by it. We further postulate that T cell abnormalities and deficiencies may increase the brain’s predisposition to damage, malfunction and epilepsy. We encourage further studies in animal models to confirm or refute these hypotheses.

### 12.2 Normal Healthy Human T Cells Are Killed *In Vitro* by Affinity-Purified GluR3B Peptide Autoimmune Antibodies of Severe Intractable Epilepsy Patients, and Also by Patient’s Purified Total IgG

We recently published an interdisciplinary and multidisciplinary article entitled: “Dual-Targeted Autoimmune Sword in Fatal Epilepsy: Patient’s glutamate receptor AMPA GluR3 **B** peptide autoimmune antibodies bind, induce Reactive Oxygen Species (ROS) in, and kill both human neural cells and T cells” (10).

In this study, we found that normal human CD3^+^ T cells of both helper CD4^+^ and cytotoxic CD8^+^ types express high levels of ionotropic and metabotropic Glutamate receptors, as do human neural cells. This was evident by expression of both AMPA-GluR3, NMDA-NR1 and mGluR3 on the cell surface of normal naive human T cells, detected by few methodologies (10).

The high expression of GluR3 in T cells revealed in this study (10) was in line with our pioneering discovery that T cells express high levels of GluR3 identical in sequence to the neural GluR3 (78), and that Glutamate itself activates several important T cell functions (78, 79, 100). These findings also corroborate the findings of GluR3 expression, function, importance and disease-associated changes in T cells of Multiple Sclerosis patients (102, 116).

A review on Glutamate and T cells (100), another one on Glutamate, T cells and Multiple Sclerosis (103), a book chapter on Glutamate in the immune system (104), and the papers cited in these publications, provide more in-depth knowledge and discussion on this topic.

Strikingly, we recently discovered that affinity-purified GluR3 **B** peptide antibodies of NS patients killed *in vitro*, on their own (without the involvement of any other factor), normal human T cells, as well as human neural cells, within a single hour (10). NS patient’s total purified IgGs, or serum also killed normal human T cells. In contrast, control affinity-purified antibodies, IgG or serum of healthy subjects did **not** kill T cells.

Whether or not epilepsy patient’s own T cells are being killed *in vivo* in their body, by their own GluR3 **B** peptide autoimmune antibodies, and whether such possible killing of patient’s T cells by their autoimmune antibodies cause mild or severe autoimmune-mediated T cell immunodeficiency in epilepsy patients, which in turn make them susceptible to infectious organisms, cancer and other health threats, are all still open questions that requires further investigations.

### 12.3 Hypothesis: Autoimmune Antibodies That Target Various Neurotransmitter Receptors Other Than Glutamate Receptors, and That Are Present in Patients With Diverse Neurological Diseases, Could Also Damage T Cells That Definitely Express These Neurotransmitter Receptors Too

The essence of this novel hypothesis is presented in **Table 3**, It is based on two different lines of evidence, which we put together herein.

First, patients with various neurological and psychiatric diseases, harbor autoimmune antibodies that target various Neurotransmitter receptors (**Table 3**).

Second, T cells, alike neural cells, express most if not all these Neurotransmitter receptors (**Table 3**), and can be affected, either activated or suppressed, directly by some of the respective Neurotransmitters. We study this scientific and clinical topic for many years, gave it the name **‘Nerve-Driven Immunity**’, and discuss it in many original papers, reviews, book, and international meetings named ‘Nerve-Driven Immunity’ [see for example (105, 106, 100, 112, 99)]. In addition, the effects of Neurotransmitters on T cells are being studied by other researches worldwide, and some exciting findings were revealed in recent years.

T cells express on their cell surface many functional Neurotransmitter receptors, among them: Dopamine receptors (107–112, 179–182, and for reviews see (111, 112, 181), Glutamate receptors (78–80, 102, 113–117), and for reviews see 100, 103, 104), Acetylcholine receptors (118, 119), GABA receptors (120, 121, 183–187), Serotonin receptors (122–125) and Adrenergic receptors (126–134).

Interestingly, partial support of our hypothesis, that T cells may be damaged in Autoimmune Epilepsy, comes from a recent relevant discovery that patients with NMDAR encephalitis, having NMDA-NR1 antibodies, have significantly lower frequencies of CD154-expressing NR1-reactive helper T cells than healthy controls, and produce significantly less inflammatory cytokines (52). Are normal T cells, which are beneficial and necessary for the proper functioning of both the immune system and the nervous system (97, 98, 101, 96, 99), indeed damaged in patients with several neurological diseases (specified in **Table 3**), as a result of the destruction of the T cells by the patient’s own autoimmune antibodies, which target the Neurotransmitter receptors which T cells express (**Table 3**), alike neural cells do? To the best of our knowledge, this novel question has not yet been addressed. The clinical bell rings now for scientific and clinical investigations of these questions.

### 12.4 Autoimmune and/or Cytotoxic Human T Cells of Epilepsy Patients Induce Profound Neuropathogenic Effects, and These Can Induce or Promote Epilepsy

The presence of autoimmune or cytotoxic T cells in the brain can cause profound brain damage, as has been demonstrated in various studies, in various neurological diseases. Several extensive studies, performed by different research groups, discovered that CD8^+^ and CD4^+^ T cells play a central role in Rasmussen’s encephalitis (RE). This pediatric Autoimmune Epilepsy is characterized by GluR3 autoimmune antibodies (135–140, 2, 4, 7, 20, 21, 23). T cells are especially evident in the intermediate stage of RE (139), during which evidence shows increased infiltrate and microglial and astrocytic reactions in a pan-laminar distribution, with indications of neuronal injury and neuronal drop out (136–139).

The infiltrating lymphocytes are mainly cytotoxic CD8^+^ T cells, fewer are helper CD4^+^ T cells, while perivascular B cells are infrequent. The CD8^+^ T cells are clonally expanded, with subpopulations being CNS restricted. This suggests local T cell proliferation in the CNS, in response to an antigen within the CNS (136). Furthermore, CD8^+^ T cells with granzyme B immunoreactivity are found in apposition to neurons and astrocytes, thus supporting their pathologic role in RE (137, 138). Based on these observations and many others not cited herein, it is clear that autoimmune and cytotoxic T cells play a central pathological role in RE, and can most probably do so in patients with other types of Autoimmune Epilepsy.

### 12.5 GluR3B Peptide T Cells Are Produced, Exhibit Skewed Pattern Of Expression of Particular TCR Vβ Families, and May Damage the Brain

A previous study of our group (7) (mentioned already in Part 4.3), yielded two interesting findings: **1**. GluR3 **B-**peptide immunized mice developed GluR3 **B** peptide-specific T cells; **2**. The T cells within the mice spleens expressed significantly biased frequencies of TCR Vβ families: Vβ11, Vβ7 and Vβ8. This skewed pattern of expression of particular TCR Vβ families suggests clonal proliferation of specific autoimmune T cell populations in the spleens of GluR3 **B**-immunized mice, in response to the GluR3 **B** peptide.

Importantly, the brain of the GluR3 **B**-immunized mice having both autoimmune GluR3 **B**-specific antibodies and T cells, was damaged. The brain damage was evident by several types of neuropathologies, that partially resembled those occurring in brains of RE patients. These include: thickening of the cerebral meninges with lymphocytic infiltrates, cerebellar cortical abiotrophy with loss of Purkinje cells, occasional gliosis, and moderate to severe spongiform degeneration of the white matter, especially in the cerebellum. The mice also expressed subclinical behavioral abnormalities (7).

Based on all these findings, we suspect that epilepsy patients may have both autoimmune GluR3 **B** peptide antibodies and T cells, and that both can damage the brain and promote epilepsy. In addition, it could very well be that patients with Autoimmune Epilepsy have T cells directed against additional autoantigens, against which only antibodies were tested and detected so far.

### 12.6 Patient’s Autoimmune Antibodies Can Cause Elevation and Accumulation of Either Beneficial or Detrimental T Cells in Several Brain Regions

We recently discovered that when purified IgG of epileptic NS patients rich in GluR3 **B** peptide autoimmune antibodies was released continuously in brains of normal mice for 1 week, this IgG caused: **1**. Seizures, **2**. Multiple brain damages, **3**. Elevation and accumulation of CD3^+^ T cells, in addition to activated microglia, in few brain regions (10).

These findings suggest that the patient's IgG that was rich in autoimmune GluR3 **B** peptide antibodies, and that reached the brain and bound neural cells (as we detected), and/or the brain damage and seizures they caused, induced recruitment and clustering of T cells in specific damaged brain regions (10). It could be that additional autoimmune antibodies of patients with Autoimmune Epilepsy can induce similar effects.

We suspect that the non-autoimmune T cells, that were recruited to specific brain loci by the patient's IgGs or by the damage they caused, could be either normal beneficial T cells recruited to the brain to assist in repair of the brain tissue damage caused by the autoimmune antibodies, or cytotoxic detrimental T cells, that augment the brain damage. Only further needed studies in animal models will be able to distinguish between these possibilities.

## 13. The HLA Molecules Are Important for Brain Development, Function, and Protection. The HLA Haplotype Can Associate With Either Susceptibility Or Protection From Autoimmune Epilepsy

The Human Leukocyte antigen (HLA) molecules are critical for initiating effective immunity against foreign microorganisms. Additionally, they contribute to proper brain development, function and protection (141–143). Yet, the HLA haplotype of an individual can also predispose to detrimental autoimmunity, and plays a role in numerous autoimmune diseases. Our recent studies show that this may be the case also for Autoimmune Epilepsy (141–143).

The important role of HLA molecules in the nervous system (141–143) seems to be unknown to many immunologists and neurologists, and therefore deserves to be mentioned briefly. HLA class I molecules are expressed by subsets of neurons in both the adult and developing mammalian brain, and play a role in synaptic plasticity, brain development and axonal regeneration (141–143). However, constitutive neuronal HLA expression can play a neuro-inflammatory role in neurodegenerative diseases. This involves pro-inflammatory cytokines, activated microglia and increased cytosolic oxidative stress. Thus, the HLA-class I molecules play an important role in both normal CNS development and function, and in some CNS diseases (142).

A previous study (143) evaluated the expression and cellular pattern of MHC-I molecules in focal glioneuronal lesions associated with intractable epilepsy. MHC-I expression was studied in epilepsy surgery cases with focal cortical dysplasia (FCD I; FCD IIa and FCD IIb), tuberous sclerosis complex (TSC, cortical tubers) or ganglioglioma (GG), using immunocytochemistry. Evaluation of T cells with granzyme B^+^ granules and albumin immunoreactivity was also performed.

The results showed that all the lesions were characterized by MHC-I expression in blood vessels. Expression in both endothelial and microglial cells as well as in neurons (dysmorphic/dysplastic neurons) was observed in FCD II, TSC and GG cases. In addition, perivascular and parenchymal T cells (CD8^+^ cytotoxic T cells) with granzyme B^+^ granules in FCD IIb and TSC specimens were observed. Albumin extravasation, with uptake in astrocytes, was observed in FCD IIb and GG cases (175). These findings indicate that a prominent upregulation of MHC-I as part of the immune response occurs in epileptogenic glioneuronal lesions. In particular, the induction of HLA-class I expression in neuronal cells appears to be a feature of type II FCD, TSC and GG, and may represent an important accompanying event of the immune response, associated with BBB dysfunction, in these developmental lesions (143).

Do HLA molecules, and especially their peptide-binding grooves, play an important role also in Autoimmune Epilepsy?

In our recently published immunogenetic study (144) we discovered that protection or susceptibility to the devastating childhood epilepsy NS, which we found to be a type of Autoimmune Epilepsy (10), associates with immunogenetic fingerprints in the HLA binding groove (144). In this study we analyzed seven HLA loci in 48 NS patients and 51 healthy controls. We discovered that NS associates significantly with both a protective HLA haplotype: HLA-B*42:01, C*17:01, DRB1*03:02, DQB1*04:02 and DQA1*04:01, and a susceptible motif: Ala24, Glu63 and Phe67, in the HLA-B peptide-binding groove (144). These amino acids create a hydrophobic and sterically closed peptide-binding HLA pocket, favoring the proline residue.

These findings suggest that immunogenetic fingerprints in HLA peptide-binding grooves tentatively associate with protection or susceptibility to NS. Accordingly, we envision that the presence of different HLA molecules, i.e. different HLA haplotype in different individuals, may explain why under similar environmental factors, only some of them, within the same families and location, develop NS and later often die, while others do not.

In our additional and most recent paper on immunogenetics and Autoimmune Epilepsy (145), we investigated the relation of NS and the functional polymorphisms in the gene coding for immunogenetic Macrophage Migration Inhibitory Factor (MIF) - an immune-regulatory cytokine playing a central role in modulating both innate and adaptive immune responses. MIF is also involved in various pathologies: infectious, autoimmune and neurodegenerative diseases, epilepsy and others. Based on these facts, we assessed two functional polymorphisms in the MIF gene, a −794 CATT 5–8 microsatellite repeat, and a −173 G/C single-nucleotide polymorphism, in 49 NS patients and 51 healthy controls (145).

We discovered that the HLA haplotype HLA-B*42:01, C*17:01, 338 DRB1*03:02, DQB1*04:02 and DQA1*04:01 has a dominant protective effect over the MIF -173 CC/CG genotypes. The later was still significantly associated with disease protection in subjects that do not carry this HLA haplotype. Furthermore, the presence of the HLA-B binding groove motif: Ala24, Glu63 and Phe67 associated with disease susceptibility in subjects that carry the MIF -173 CC/CG genotypes (145). These findings suggest that the HLA susceptibility or protective effect are stronger than the MIF effect in NS. Nonetheless, the MIF -173C allele was found to be significantly associated with disease protection (145).

Taken together, all the immunogenetic findings presented in this chapter, indicate that the HLA haplotypes can play an important direct and indirect role in Autoimmune Epilepsy, and call for further studies on this topic, on a large cohorts of patients with intractable Autoimmune Epilepsy, that have different types of autoimmune antibodies.

## 14 Therapy-Related Findings That Inspire Hope: Several Treatments Can Decrease Seizures in Patients With Autoimmune Epilepsy

Patients with Autoimmune Epilepsy or with encephalitis and seizures, can benefit from various therapeutic strategies, as described and discussed in several original papers and reviews [see for example (17, 18, 8, 38–43, 148, 11, 12, 45, 53, 57, 60, 61, 64, 69, 71, 76, 88, 146, 148) and papers cited in these papers]. Therefore, the topic does not necessitate repetition herein, only a brief summary. The readers interested in learning more, are referred to articles on this topic, among them those cited above.

Overall, immunotherapy administered early after the first diagnosis of Autoimmune Epilepsy seems to be particularly effective, and few optional therapeutic strategies exist, either as a monotherapy or a combinatorial therapy. These therapeutic options can be classified into three therapeutic groups.

**Group 1**. **Immunosuppressive and anti-inflammatory chemical and biological drugs**: Intravenous Immunoglobulin (IvIg) Methylprednisolone, Cyclophosphamide, Tacrolimus, Natalizumab - monoclonal antibody (mAb) against cell adhesion molecule α4-integrin, Rituximab – mAb against CD20, expressed primarily on B cells, and Adalimumab - mAb against TNFα;

**Group 2. Immune purging procedures**: IgG absorption and plasmapheresis.

**Group 3. Some AEDs, or combination of AEDs with immunotherapy**.

For more in depth information on therapy of Autoimmune Epilepsy, the readers are referred to the clinical reviews focusing on this important topic (17, 18, 8, 38–43, 148, 11, 12, 45, 53, 57, 60, 61, 64, 66, 69, 71, 76, 88, 146, 148).

## 15. Concluding Remarks

**Figure 3:**
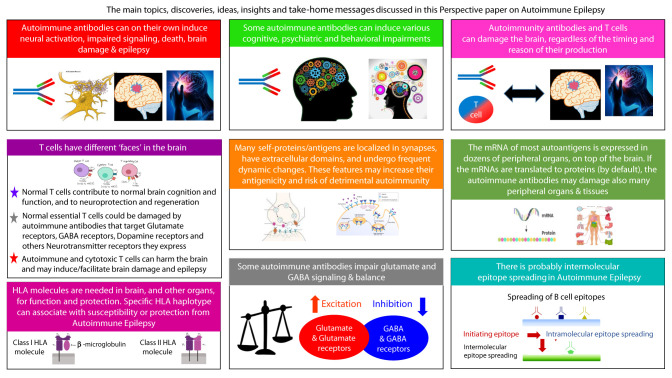
The main topics, discoveries, ideas, insights and take-home messages discussed in this Perspective paper on Autoimmune Epilepsy.

The main topics, discoveries, insights, ideas and take-home messages discussed in this Perspective paper, are summarized very concisely, in single sentences and graphical symbols in **Figure 3**. Most of them are also summarized briefly in the Abstract of this paper.

We conclude this article in the hope, that the findings, analyzes, interpretations, novel ideas, hypotheses and recommendations raised, reviewed and discussed in this article will encourage new thoughts and scientific and clinical research. We also hope they will contribute to new understanding, and lead to new practical actions, that will benefit epilepsy patients, and also physicians, scientists, and students.

## Author Contributions

The authors confirm being the only contributors of this work, and approve it for publication.

## Conflict of Interest

The authors declare that the research was conducted in the absence of any commercial or financial relationships that could be construed as a potential conflict of interest.

## Publisher’s Note

All claims expressed in this article are solely those of the authors and do not necessarily represent those of their affiliated organizations, or those of the publisher, the editors and the reviewers. Any product that may be evaluated in this article, or claim that may be made by its manufacturer, is not guaranteed or endorsed by the publisher.
